# Forcing Versus Feedback: Epidemic Malaria and Monsoon Rains in Northwest India

**DOI:** 10.1371/journal.pcbi.1000898

**Published:** 2010-09-02

**Authors:** Karina Laneri, Anindya Bhadra, Edward L. Ionides, Menno Bouma, Ramesh C. Dhiman, Rajpal S. Yadav, Mercedes Pascual

**Affiliations:** 1 Department of Ecology and Evolutionary Biology, University of Michigan, Ann Arbor, Michigan, United States of America; 2 Department of Statistics, University of Michigan, Ann Arbor, Michigan, United States of America; 3 Fogarty International Center, National Institutes of Health, Bethesda, Maryland, United States of America; 4 Department of Infectious and Tropical Diseases, London School of Hygiene and Tropical Medicine, University of London, London, United Kingdom; 5 National Institute of Malaria Research, Delhi, India; 6 Howard Hughes Medical Institute, University of Michigan, Ann Arbor, Michigan, United States of America; Imperial College London, United Kingdom

## Abstract

Malaria epidemics in regions with seasonal windows of transmission can vary greatly in size from year to year. A central question has been whether these interannual cycles are driven by climate, are instead generated by the intrinsic dynamics of the disease, or result from the resonance of these two mechanisms. This corresponds to the more general inverse problem of identifying the respective roles of external forcings vs. internal feedbacks from time series for nonlinear and noisy systems. We propose here a quantitative approach to formally compare rival hypotheses on climate vs. disease dynamics, or external forcings vs. internal feedbacks, that combines dynamical models with recently developed, computational inference methods. The interannual patterns of epidemic malaria are investigated here for desert regions of northwest India, with extensive epidemiological records for *Plasmodium falciparum* malaria for the past two decades. We formulate a dynamical model of malaria transmission that explicitly incorporates rainfall, and we rely on recent advances on parameter estimation for nonlinear and stochastic dynamical systems based on sequential Monte Carlo methods. Results show a significant effect of rainfall in the inter-annual variability of epidemic malaria that involves a threshold in the disease response. The model exhibits high prediction skill for yearly cases in the malaria transmission season following the monsoonal rains. Consideration of a more complex model with clinical immunity demonstrates the robustness of the findings and suggests a role of infected individuals that lack clinical symptoms as a reservoir for transmission. Our results indicate that the nonlinear dynamics of the disease itself play a role at the seasonal, but not the interannual, time scales. They illustrate the feasibility of forecasting malaria epidemics in desert and semi-arid regions of India based on climate variability. This approach should be applicable to malaria in other locations, to other infectious diseases, and to other nonlinear systems under forcing.

## Author Summary

Malaria epidemics can exhibit pronounced variation from year to year that can be driven by external forcings, such as climate, or can be generated instead by dynamic feedbacks within the disease system itself. For example, levels of immunity in the population (or control efforts) can rise and fall as the result of past levels of infection. This type of feedback is found in the dynamics of all (nonlinear) biological systems. Feedbacks can interact in complex ways with external drivers, for example by creating refractory periods. It remains a challenge to identify internal feedbacks vs. external forcings from available temporal records of aggregated reported cases and forcing variables. We propose a quantitative approach that can statistically compare the hypotheses of feedbacks vs. forcings (epidemiological vs. climate) based on dynamical and mechanistic models. Our approach is computational, based on a large number of computer simulations of the different models. We illustrate and apply the approach to the analysis of extensive monthly records for malaria incidence in desert regions of India that span two decades. Our analyses confirm the strong role of rainfall, and quantify this effect with transmission model(s) for malaria that include rainfall and are shown to exhibit a remarkable prediction skill.

## Introduction

Epidemic or ‘unstable’ malaria occurs in areas of marginal environmental conditions for the development of the parasite and the population dynamics of the mosquito vector, at the edge of the distribution of the disease. Millions of people live in the highlands and desert fringes around the tropics in Africa, Asia and South America. It is in these regions, where temperature or rainfall limit transmission, that climate variability and climate change have the potential to most strongly impact the population dynamics of the disease. Determining the role of climate variability is fundamental to evaluate both the feasibility of early-warning systems for infectious diseases based on climate, as well as the consequences of longer-term trends in climate. The intermittent nature of epidemics in unstable malaria regions results in populations that cannot sustain high levels of acquired immunity and are therefore susceptible to high morbidity and mortality; it also poses a different challenge for control efforts than the more stable, high-transmission intensity, endemic regions [1]. The ability to forecast and identify epidemic events becomes one important component of control efforts that can contribute to the timely implementation of effective prevention and treatment, as recognized by on-going efforts to develop malaria early-warning systems (MEWS) [1]–[3].

Studies of the role of climate variability, not just in malaria but also in other infectious diseases, have been limited by the scarcity of long temporal records of disease incidence, and by the difficulties of addressing the role of climate forcing in the context of the nonlinear dynamics of infectious diseases. These systems are well-known to behave as seasonally-forced nonlinear oscillators, capable of generating substantive variation from year to year on their own, in the complete absence of any year-to-year variation in an external driver such as climate [4]–[7]. The waxing and waning of immunity in the population has long been recognized as the key mechanism behind this intrinsic ability of disease systems to cycle on characteristic time scales longer than one year, leading to inter-annual variation in the size of outbreaks. The severity of epidemics is influenced by the size of the non-immune population, which in turn decreases as levels of infection rise, and rebuilds with the loss of functional immunity and demographic processes such as birth and immigration [8], [9]. This dynamic feedback within the disease system underlies different conclusions on the role of climate variability in the inter-annual variation of vector-borne diseases, especially for malaria in E. African highlands [7], [10]–[13]. It can also modulate the sensitivity of the response to climate drivers by generating periods of time that are refractory to forcing because of the temporary depletion of the non-immune population that fuels transmission [14]. Other feedbacks such as those generated by control efforts that are reactive to previous levels of infection, or behavioral responses, can also generate interannual cycles and refractory periods.

Extensive epidemiological records for the past two decades in desert and semi-arid districts of India provide an opportunity to examine the role of rainfall on malaria epidemics of *Plasmodium falciparum*, while also taking into account disease dynamics. The periodically recurring epidemics in the (semi) arid parts of India, particularly the Punjab, were amongst the most devastating described in the history of malaria [15]. Early efforts to understand and forecast malaria epidemics included not just rainfall but also spleen rate, the proportion of children with enlarged spleens, which reflected recent exposure and provided an indirect measure of population levels of immunity [16]–[18]. After the malaria eradication efforts in India were abandoned in the 1970s, the epidemic belt shifted to the more arid and increasingly populous regions of Gujarat and Rajasthan. In the last decades the severity of these epidemics have made developing a MEWS based on rainfall [19] and rainfall forecasts [20] a public health priority. Quantifying the role of climate variability, and doing so in the context of epidemiological dynamics, remains an important open problem for these regions and for epidemic malaria in general. Recent developments on parameter estimation for nonlinear dynamical systems now make possible the consideration of epidemiological models that can be confronted to noisy and incomplete data [21], [22]. We show here that these recent developments provide a basis for a formal statistical comparison of rival hypotheses on extrinsic drivers vs. intrinsic feedbacks, or more specifically, on climate vs. nonlinear disease dynamics, represented in mechanistic dynamical models. This differs from previous efforts to answer this same question, that did not provide a formal statistical comparison of hypotheses, based on models whose structures were constrained to simple forms by the inference methods, and that did not include the climate covariate explicitly (e.g. [10], [14]). We formulate here a dynamical model of malaria transmission that incorporates rainfall explicitly. The sequential Monte Carlo methods allow for more flexible representations, as well as the consideration of process and measurement noise, because their computational implementation only requires the numerical simulation of the dynamical models. Desert malaria provides an ideal initial application of this approach, given previous correlative evidence for an association between desert malaria and rainfall from shorter records in Africa [9], [23], and the potentially-weak dynamical role of immunity in these epidemic regions at the edge of the geographical distribution of the disease.

Our analysis shows a strong and significant effect of rainfall in the inter-annual variability of epidemic malaria that involves a threshold in the disease response. Simulations of the transmission model for twenty years capture remarkably well the observed epidemic patterns when rainfall is prescribed. We demonstrate the high prediction skill of the model for yearly cases in the transmission season following the monsoonal rains. We compare this skill to that of statistical models, in particular a mixture model that incorporates a threshold, to examine the feasibility of forecasting epidemics in these regions with simpler, more phenomenological, models of malaria's response to rainfall. We end by showing the robustness of the results to a more complex transmission model that includes clinical immunity, and the value of the dynamical models for predicting the time course of epidemics.

## Results

**Figure 1:**
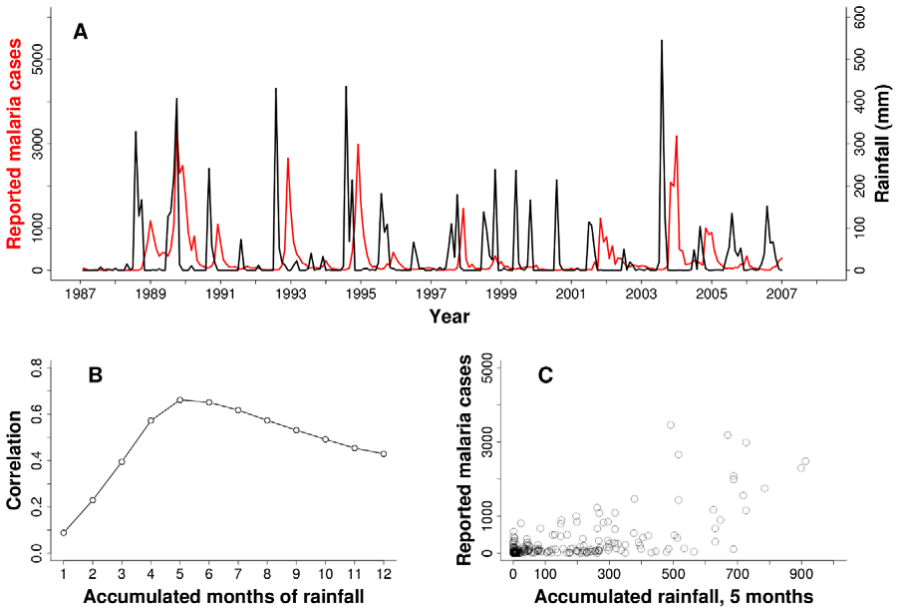
Malaria cases and rainfall. (A) Monthly 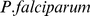 malaria reported cases (red) and monthly rainfall from local stations (black) for Kutch. (B) Correlation between accumulated rainfall in different time windows preceding the month of reported cases. A maximum is observed when rainfall is accumulated over 5 to 6 months. (C) Monthly reported cases as a function of accumulated rainfall in the previous five months. A threshold, nonlinear response is apparent with no effect of rainfall below a value of around 200mm and an increase in both the mean and the variance of cases above it.

**Figure 2:**
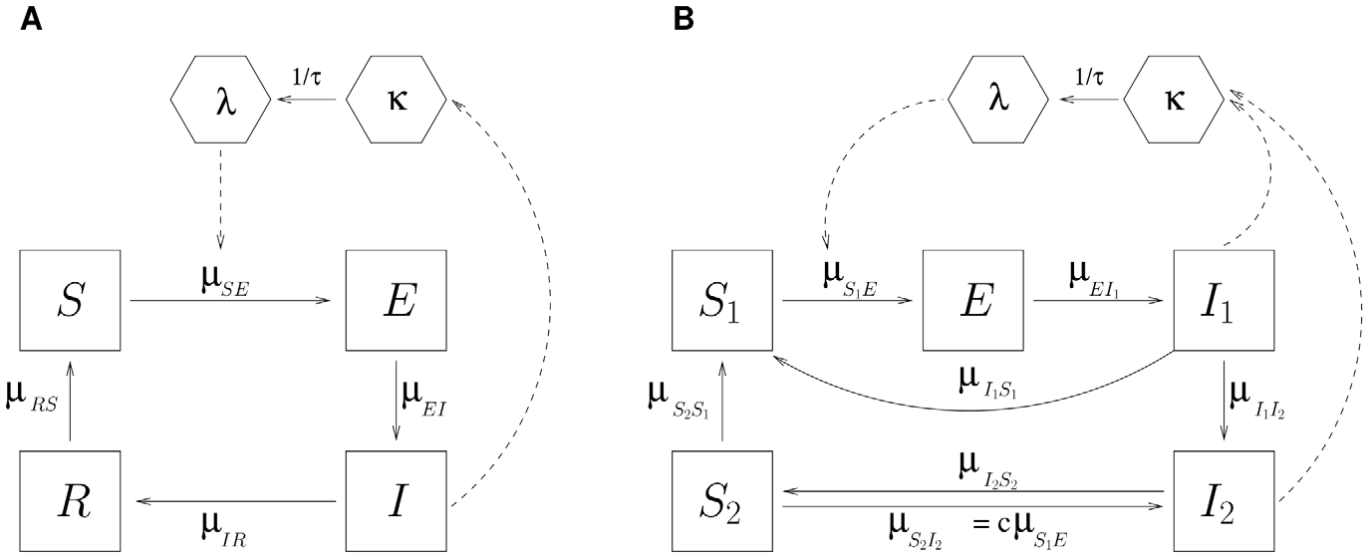
Flow diagram for two compartment models of malaria transmission. (A) shows the VSEIRS model and (B) shows the 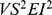 model. Human classes in (A) are 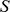 (Susceptible), 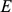 (Exposed), 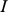 (Infected), and 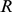 (Recovered). Mosquito classes are *κ* (latent force of infection) and *λ* (current force of infection). The possibility of transition between classes 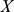 and 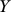 is denoted by a solid arrow, with the corresponding rate written as 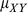. The average time of mosquitoes in the latent state is denoted by 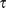. The dotted arrows represent interactions between the human and mosquito stages of the parasite. The model in (B) adds clinical immunity [25], by differentiating between clinical infections that contribute to the measured cases, and less severe infections in a new class 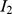 that are not clinical but remain infectious to mosquitoes at a lower level than 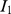. Clinical infections can fully recover becoming susceptible again, or remain parasitemic and transition to 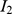. Recovery from mild infections results in individuals who are fully protected from clinical disease, in class 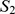, whose further exposure to infected mosquitoes, can result again in mild infections. In time, clinical immunity can also be lost, with transitions from 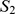 to 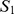, and therefore the return to full susceptibility. Only a fraction 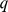 of individuals in 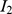 contribute to the force of infection; the susceptibility to infection is reduced by a factor 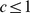 in class 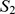 relative to 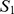.

A maximum correlation between monthly cases and rainfall was found when rainfall was accumulated for the five to six previous months (Figure 1B and Figure S3C). A nonlinear response of cases to accumulated rainfall is evident in Figure 1C, with no apparent response of the disease below a threshold of approximately 200 mm, and an increase in both cases and variability above this threshold. Based on these observations, we incorporated accumulated rainfall and a threshold response in the force of infection of a model of malaria transmission (Methods and Figure 2), where both the value of the threshold and the length of the relevant time window previous to reported cases are parameters to be estimated.

Our models for *P. falciparum* malaria were developed to capture some key aspects of the human, parasite and vector dynamics while remaining sufficiently parsimonious for parameters to be estimated directly from the available time series data. The structures of the two models are shown in Figure 2 and their formulation described in the Methods and in Text S1. We begin with the simpler model, with the simpler representation of waning immunity, in which immune individuals are temporarily protected from both (clinical) disease and infection. We follow with a more realistic representation of malarial immunity in which we differentiate susceptibility to disease from that to infection [24]–[26].

**Figure 3:**
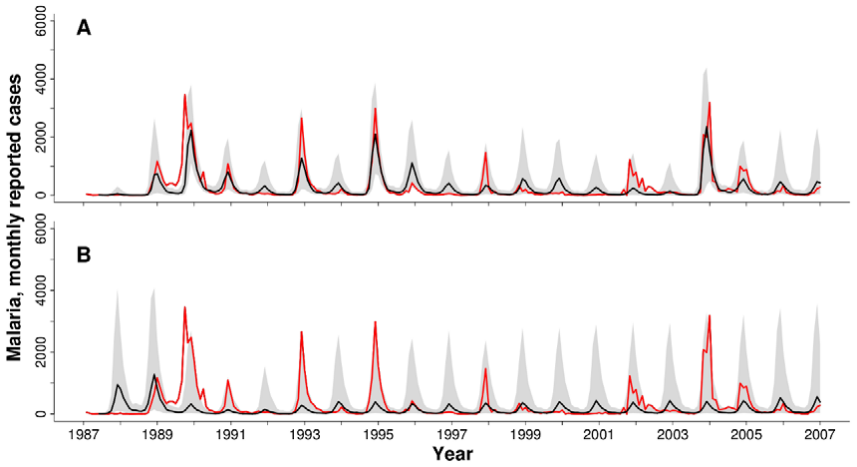
Reported monthly malaria cases and simulations for Kutch. Black lines show the median of ten thousand simulations; the shadowed regions correspond to the range between the 10% and 90% percentiles of the simulations. Red lines show the reported cases. (A) VSEIRS model with rainfall; (B) VSEIRS model without rainfall. Note that these curves do not represent the fit of the model one time-step ahead but the numerical simulation from estimated initial conditions at the end of 1986 for the complete twenty years' period, using observed rainfall values.

The VSEIRS model that includes rainfall in the force of infection performs better than the model without rainfall, based on log-likelihoods (respective log-likelihoods for Kutch are −1265.0 and −1275.0; 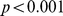, chi-square likelihood ratio test). We can also compare these values to those obtained for a linear seasonal autoregressive moving-average models (SARIMA) with and without rainfall. These comparisons are meant only as a general point of reference: if our mechanistic models fit better, or at least not substantially worse, than flexible statistical models then we conclude that they are capable of explaining most features of the available time series data. Both disease models, with and without rainfall, are better supported by the data than their SARIMA counterparts (SARIMA 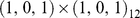, respective log-likelihoods are −1322.6 and −1329.0) (Table S1). This is also the case if we base this comparison on the Akaike Information Criterion (AIC) to take into account model complexity and penalize the likelihood based on the number of parameters (Table S1). Figure 3A shows numerical simulations of the VSEIRS model that includes rainfall together with the malaria data. This model captures well the observed patterns of the epidemics, in particular the pattern of large outbreaks followed by smaller ones, with exceptions during 1999–2001 (including the large earthquake of 2001). The similarity is striking given that these simulations are not next-step predictions, but predicted trajectories for the whole twenty years starting only from estimated initial conditions in 1987. When rainfall is not included, simulations of the VSEIRS model (Figure 3B) have a poor resemblance to the data, and generate interannual cycles of approximately 5 years. Similar results were obtained for the district of Barmer and are shown in Text S1 and Figure S5. In particular, Barmer experienced an extremely large epidemic, of nine thousand reported cases, in 1994–1995, coincident with an extreme in precipitation (Figure S3A and Figure S3B). This epidemic is five times larger than any of the outbreaks in the data, and makes the fitting of the models more challenging as reflected in likelihoods comparable to those of SARIMA (with and without rainfall respectively) (Table S1).

One difference between the parameters of the two VSEIRS models is the duration of ‘effective’ immunity at the population level (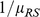 in Table 1). Immunity is shorter when rainfall is included, lasting approximately 2 months, instead of 8 years without rainfall, in the respective models with the best likelihoods. Despite fairly broad confidence intervals, some interesting patterns are apparent in the profiles of this parameter (Figure S6). In particular, the profile for the model with rainfall is bimodal which gives rise to a discontinuous confidence region, including short durations of immunity (below one year, corresponding to the maximum likelihood estimate, MLE), and longer durations around five years. This second peak maps approximately to the MLE of the model without the climate covariate. In other words, the model with rainfall fits the data in two different ways: for short immunity, interannual variability would be mostly driven by rainfall, given that the depletion of susceptibles is short-lived and within the epidemic season; whereas for longer-lasting immunity, both rainfall and the nonlinear intrinsic dynamics of the system would play a role. The corresponding log-likelihoods of these two solutions (−1265.0 and −1266.8, respectively) suggest that the short and effectively negligible duration of immunity provides a better explanation for the data, though both modes, since they fall in the confidence region, are statistically consistent with the data. To compare these further, we examine the resulting dynamics of the cases from the perspective of the dominant periodicities using wavelet analysis (see [27], [28] for a description of this method in the context of population dynamics).

**Table 1:** Selected point estimates for the VSEIRS model with and without rainfall.

	VSEIRS with rain (years)	VSEIRS without rain (years)
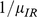	0.026	0.073
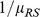	0.176	8.621
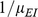	0.095	0.137
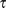	0.033	0.025

A full list of all estimated parameters is given in Table S3.

Figure S12 shows that the wavelet spectrum of the cases corresponds closely to that of rainfall, with the variability at a period of one year reflecting seasonality and showing the timing of large annual epidemics. Variability is also apparent transiently at a number of longer periods, including values of approximately 3 years, especially in the first decade and after 2002, between 4 and 5 years from 1990 to 2000, and two years from 2000. This similarity supports the role of rainfall variability as a main driver of the dynamics of cases, and a response of the system that does not involve the nonlinear dynamics of the disease itself. Representative wavelet spectra for simulations with the three models with the best likelihoods support this conclusion. These are shown in Figure S13, for the model without rainfall, and the model with rainfall but with the two different lengths of immunity. When rainfall is not included, the model tends to generate cycles of periods from four to five years, and large epidemics whose timing does not match that of observations (see regions of high power at period one in the wavelet spectrum Figure S13 A and compare with Figure S12 B). Another feature that compares poorly with the data are the long intervals with no variability for periods between one and two years that follow large epidemics. These refractory intervals during which there is little signal, even at seasonal time scales, result from the relatively long duration of immunity which reduces the pool of susceptibles after large events. When rainfall is added in the model with similarly long immunity, the timing of epidemics improves but the existence of these refractory intervals persists (Figure S13 C). Finally, simulations from the model with rainfall and a negligible duration of immunity are better able to capture both the timing of the large (annual) events, and also many of the interannual features of the variability, in terms of timing and periods (compare the spectra in Figure S12 B and Figure S13 B).

To further explore the possibility of an interaction between the longer immunity and rainfall forcing, we considered an intermediate model, with the epidemiological parameters fixed at the values of the best VSEIRS model without rainfall (see Text S1). Only the rainfall and noise parameters were then fitted to the data. This intermediate model was unable to reproduce the inter-annual oscillations observed in the reported temporal series (Figure S9).

Another parameter of interest is the delay between the latent and current force of infection. This was estimated to be approximately 10 days in the model without rainfall (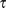 in Table 1) and around 11 days when rainfall is included (Table 1; Figure S8). These values are consistent with empirical values of the parasite's development given the observed temperatures in these regions. In this case, the delay represents the combined effect of multiple processes that establish a lag in transmission, relative not only to previous levels of infection but also to precipitation, such as the development of larvae into adult mosquitoes. The value of the delay will further depend on the specific variable chosen to represent rainfall's influence; here, accumulated rainfall in the previous five months. Another parameter estimate of interest is the reporting rate which was found to be low, with a small fraction of the infected population detected by the surveillance system in the two districts (see 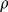 in Table S2, Table S3, Table S4 and Figure S7).

The performance of our best VSEIRS model suggests the feasibility of forecasting malaria cases as a function of previous rainfall. Moreover, the short duration of immunity, which here involves full protection to both disease and infection, further suggests that the depletion and replenishment of susceptibles, that is at the heart of intrinsic interannual cycles in infectious disease dynamics, does not play an important role here, despite the low reporting rate. This is confirmed by simulations with a fixed and large value of 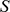 that display essentially the same patterns as those of the best-fit model. Hence, the nonlinear dynamics of the disease in this model are not crucial for the interannual cycles and simpler statistical models may also perform well for forecasting cases aggregated for the whole season. We considered both a standard linear regression model and a mixture model that effectively implements a threshold response to rainfall and an increasing variance as a function of rainfall (Figure 1C) (see Methods and Text S1 for details on the statistical models and measures to evaluate predictions). For Barmer, the VSEIRS model with rainfall exhibits the best performance overall, regardless of how we measure prediction performance, with the mixture model second in terms of prediction likelihoods and with fairly high values of prediction skill for both models (Table 2). For Kutch, high values of prediction skill are also found especially for the VSEIRS model with rainfall (Table 2). Prediction likelihoods place the VSEIRS model with rainfall on top (Table 2). Overall, the VSEIRS model without rainfall exhibits a very low predictive ability for Kutch, as expected. This is not so for Barmer, possibly because the impact of rainfall is strongly concentrated in one single extreme event, for the extreme rains of 1994.

**Table 2:** Hindcast prediction performance.

	Kutch			Barmer		
	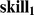	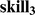	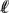	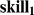	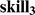	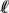
1. VSEIRS with rainfall	0.899	0.798	−161.3	0.928	0.887	−141.0
2. VSEIRS without rainfall	0.545	−0.921	−176.2	0.747	0.596	−150.0
3. Linear model	0.787	0.619	−176.7	−0.004	0.405	−181.9
4. Mixture negative binomial model	0.753	0.409	−167.3	0.826	0.613	−147.6

Subscripts 1 and 3 denote the model whose variances were used for the calculation of the skill measure. 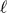 is the prediction log likelihood as defined in Text S1.

**Figure 4:**
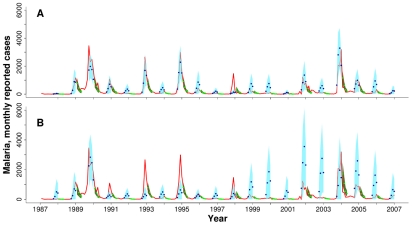
Hindcast predictions for the time course of epidemics for Kutch. The malaria data are shown in red. Superimposed on these observations, we show the predicted mean cases from one to four months ahead obtained by simulating the VSEIRS model from (1) the end of August (blue dots) and (2) the end of December (green dots). Shadowed regions in respective colors correspond to the standard deviation from a set of 5000 predicted values for each given time. Notice that this procedure requires the estimation not only of the observed state (i. e. reported cases) but also of all non-observed states at each time (i.e. S,E,I,R,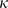,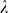). Simulations of the model require the accumulated rainfall in the previous five months: to obtain this quantity, the observed rainfall is used only until the initial time (end of August or December) and the rest of the months are completed by replacing the ‘missing’ rainfall value (given that we are predicting one to four months ahead) by its monthly average. (A) VSEIRS model with rainfall; (B) VSEIRS model without rainfall.

We have considered above forecasts of the total accumulated cases for the whole epidemic season. It is also of interest to predict the time course of cases during the epidemic season. The dynamical model of transmission with rainfall appears valuable for this purpose. This is illustrated by Figure 4 with hindcast predictions from one to four months ahead, for the rise of the peaks, immediately after the monsoons at the end of August, and for their decay, after the typical timing of the main peak, at the end of December.

We examined the robustness of our results by considering the data for Kutch and the more complex 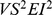 model, in which individuals can acquire clinical immunity and contract infections that lack severe symptoms (and do not contribute to reported cases) but retain the ability to transmit to mosquitoes. This formulation explicitly differentiates susceptibility to disease from that to infection, and does not allow for full immunity to both disease and infection. Interestingly, the likelihood of this model when rainfall is included (−1251.0) justifies the added complexity (Table S1). Also, this model performs better than its counterpart without rainfall (−1261.1) (see Table S1), as reflected by the substantial decrease in the levels of process noise (Table S2 and Table S3). This performance is also evident in a high prediction skill (90%) and prediction log-likelihood (−161.3) (Table 2 and Figure S11). The prediction performance of the model can be further evaluated by defining an epidemic (or extreme event) based on a threshold for the accumulated total number of cases during the epidemic season. We can then quantify the forecasted probability of an epidemic and compare this to the observed occurrence of large outbreaks (Table 3). Simulations of the 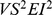 model (and the resulting medians for the total cases in the epidemic season) correctly predict four out of five epidemic years, with 2 false positives and one false negative (Table 3). Despite the higher likelihood of this model and its predictive ability, several parameters are poorly identified, as shown by their large confidence intervals, in particular the parameters determining the duration of the classes associated with clinical immunity (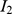 and 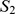) (Table S3). By contrast the estimate of the delay 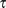 in the force of infection improves, with the confidence interval reduced to (6.2–28.4) days with the best likelihood corresponding to a value around 12 days (Figure S10). The wavelet spectrum of simulations generated with this model closely resemble that of the cases, as illustrated with a representative simulation in Figure S12 A.

**Table 3:** Hindcast predictions for malaria outbreaks during the epidemic seasons with the 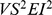 model with rainfall.

Year	Quantiles from simulation					Aggregated observed cases (Sep–Dec)	Forecast probability of an epidemic
	0.1	0.25	0.5	0.75	0.9		
1987	39	65	113	203	328	78	0.000
1988	1017	2102	4230@	6673	8740	2182	0.665@
1989	7644	8805	**10356**	12030	13748	**9990**	**1.000**
1990	496	763	1267	1949	2737	2093	0.101
1991	251	401	650	1050	1547	275	0.010
1992	1517	2500	**3902**	5293	6495	**4691**	**0.714**
1993	300	447	704	1095	1565	427	0.009
1994	1989	2942	**3987**	5023	6049	**6109**	**0.790**
1995	300	474	747	1172	1688	984	0.009
1996	124	188	301	471	702	198	0.000
1997	139	221	361	607	957	2320	0.002
1998	425	697	1170	1895	2732	837	0.100
1999	508	815	1324	2180	3159	324	0.153
2000	205	342	612	1068	1742	89	0.027
2001	1306	2036	3300@	5025	6690	2692	0.601@
2002	670	1056	1730	2784	3945	425	0.258
2003	6242	7594	**9196**	10986	12911	**7372**	**0.995**
2004	581	880	1350*	2012	2778	**2857**	0.107*
2005	425	651	1070	1708	2550	675	0.076
2006	238	395	681	1155	1795	661	0.031

The second through fifth columns show the quantiles of 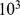 simulated epidemics aggregated over September to December in each year, using initial conditions and rainfall covariates based on information available up to August. The column ‘aggregated observed cases’ shows the reported malaria cases accumulated over the same period. We declare a year to be epidemic if cases rose to more than the 75th percentile of aggregated observed cases ( = 2733) (bold black). We forecast an epidemic if the median of the prediction distribution exceeds this threshold. Equivalently, we can measure the forecast probability of exceeding this epidemic threshold (last column), predicting an epidemic if this probability exceeds 0.5. Four out of five epidemics were forecasted correctly (bold black). The false negative (underlined *) and two false positives (underlined @) all contain the actual presence/absence of an epidemic within their central 80% prediction intervals.

**Figure 5:**
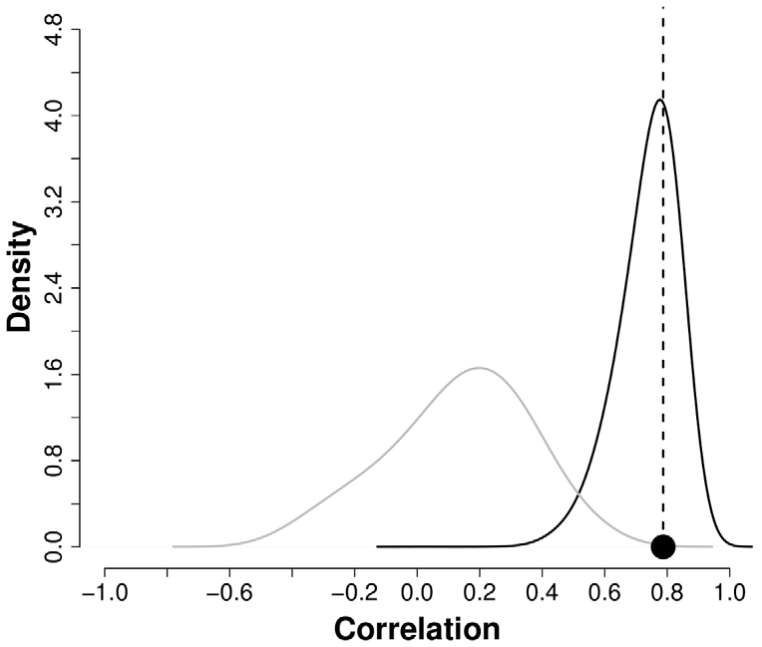
Density plot of correlation between the accumulated rainfall from May to August and the accumulated cases from September to December in 10,000 simulations from a set of 2,000 solutions for the 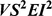 model with rainfall (black) and without rainfall (gray). The vertical line is the observed correlation of 0.778. For the model with rainfall, 38.63% of the simulations have a correlation with rainfall above the observed value (black circle).

Finally, to further compare the temporal patterns generated by this model to the data in a way that accounts for the uncertainty in the parameters, we considered the correlation value between total rainfall, accumulated during the monsoon season, and the cases, accumulated during the epidemic season. This value is influenced by both the timing and the interannual variability of the peaks in cases. We simulated repeatedly from the fitted model by resampling the 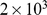 sets of plausible parameters identified while investigating the parameter space, with probabilities according to their likelihood. For each simulation, we computed the predicted correlation and generated in this way a distribution for this statistic for the two models (the 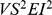 model with and without rainfall). Figure 5 shows the comparison of the observed correlation to the two distributions of correlations from simulations of these models. Whereas the distribution of the model without rainfall results in a very low probability of the observed association (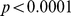), its counterpart for the model with rainfall peaks at the observed correlation (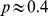). This further supports rainfall as the main driver behind the temporal variability of cases, a conclusion that is robust to the uncertainty in parameter values. A more detailed comparison of the models will be reported elsewhere [29]. We discuss below the implications of these findings.

## Discussion

We have proposed a computational approach to formally compare hypotheses on the respective roles of extrinsic forcings vs. intrinsic feedbacks in dynamical systems for which time series data are available but provide only a partial measurement of the relevant variables. This extends previous efforts on this inverse problem by incorporating climate covariates explicitly in dynamical models that also include both measurement uncertainty and dynamic noise. Our approach complements time series methods based on phenomenological, autoregressive models, developed to address the role of covariates and density-dependence in ecology and epidemiology (e.g. [30]–[32]). Concerns on the limitations of (linear) correlative analyses to infer the role of climate drivers in the interannual variability of malaria and other infectious diseases [33] can be addressed directly by formulating different hypotheses as epidemiological models. Beyond a better understanding of temporal disease patterns, such models can also contribute valuable tools for forecasting purposes. Ultimately, any early-warning system should benefit from an ensemble of models, including epidemiological models driven by climate variability.

We have applied the proposed approach to demonstrate the impact of rainfall on the interannual cycles of epidemic malaria in desert regions of India, by incorporating a climate variable into a stochastic dynamical model of disease transmission. Our approach directly confronted different hypotheses on the population dynamics of the disease based on time series data. The findings on the predominant role of rainfall are robust to consideration of more complex epidemiological models of malaria transmission with a different representation of immunity [25]. The same approach should be applicable to other nonlinear systems and to other vector-transmitted diseases in particular, including those for which the nonlinear population dynamics of the disease is especially relevant. One example is dengue for which evidence of a temporal association with the El Niño Southern Oscillation (ENSO) differs across geographic location and study [34], [35], consistent with the complex multi-strain dynamics of the disease [36]. For malaria, the role of climate variability in East African highlands could be revisited, given previous conflicting evidence in these areas where the interplay of immunity and climate is likely [7], [9], [10], . Even in more ‘stable’ malaria regions with seasonal behavior, the question of interannual climate variability might be of relevance and worth examining in retrospective records.

Our results with the simpler model suggest that immunity, in the sense of complete protection to both infection and disease, is of short duration and negligible at the population level in these semi-arid regions. These regions are expected to lie at one extreme of continuum in the gradient from stable to unstable, or endemic vs. epidemic. In this case, climate variability and not epidemiological dynamics are expected to be the main driver, as proposed for desert fringes in Africa [9], [38]. We have used, however, the term ‘effective’ population immunity to emphasize that our results apply to the dynamical role of (full) immunity at the spatial scale of districts, and do not necessarily imply that individual immunity is also short. Disease risk may be, for example, spatially heterogeneous within districts. We have tested this possibility by considering a reduced total population of susceptibles, systematically lower than that of the whole district, but obtained similar results regarding immunity and rainfall. In a more complex situation, high risk areas may shift their location from year to year, tending to mask the effect of immunity at the more aggregated spatial level of districts. Detection of immunity patterns in this case would require analyses and data at higher spatial resolutions; although they might still be effectively inconsequential at the aggregated level of districts. The bimodal character of the likelihood surface with two peaks, corresponding respectively to two different durations of immunity, raises the possibility that longer time series with a higher number of interannual cycles may support an interaction of rainfall with the nonlinear dynamics of the disease (via the longer-lasting immunity). However, the analyses of the patterns of variability generated by the model for these two peaks in parameter space do not support this conjecture. Additional data could be considered in future work by identifying multiple districts, or different areas within districts, for which it is sensible to fit more than one time series simultaneously.

Our second model follows naturally from the result that immunity as included in a typical SEIRS framework, meaning protection against disease and infection, appears negligible. The better performance of the model with clinical immunity indicates that a more complex representation of the epidemiological dynamics is warranted, and further suggests the importance of the contribution of asymptomatic infections to transmission, especially as a reservoir during the low season. In particular, the incorporation of two pathways, associated with more than one time scale of the recovery to disease susceptibility, appears warranted. This better performance is consistent with more detailed models of malaria transmission in the literature that consider the different effects or underlying mechanisms of immunity in malaria (e.g. [24], [26], [39]). These are typically confronted to age prevalence curves especially for endemic regions; it is of interest here, that time series patterns per-se also support a more complex structure of immunity than that typically used for childhood diseases, and do so for an epidemic region. Long time series patterns for malaria have not been analyzed before from this perspective; they are of particular relevance in epidemic regions where the interannual variation is high. It is also of interest to note that most mathematical arguments on the role of intrinsic disease dynamics in the interannual variability of epidemic malaria (in highland regions) rely essentially on analytical approaches related to dominant or resonant frequencies of SEIRS-type models (e.g. [7], [13]). The understanding of intrinsic interannual cycles in infectious diseases in general is influenced by the rich literature on this subject in childhood diseases that confer full immunity (see [40]). The biology of malaria and our results here suggest that such understanding is not likely to simply transfer in a relevant way. The consideration of epidemiological models in the analysis of population-level time series provides a natural link to, and opens the door for, analytical approaches [13], [41] to malaria's cycles based on empirical patterns.

However, we are here at the limit of model complexity that retrospective data on a single epidemiological variable (the number of cases) can support, as shown by the poor identifiability of specific parameters, especially those associated with the duration of clinical immunity. This is not surprising given the obvious trade-offs between epidemiological parameters that become possible in this more complex model: for example, individuals may remain for shorter times in the infectious but clinically immune class if we increase their contribution to the force of infection. Despite this limitation, it is interesting that even for an epidemic region, where transmission is intermittent and at its limit for disease persistence, we can clearly improve the fit of the data by including asymptomatic infections. The low estimate of the reporting rate also suggests the possibility of a considerable number of asymptomatics and a larger disease burden than reflected by the detected cases. Microscopy has been shown to underestimate the diagnosis of malaria compared to PCR (polymerase chain reaction) in similarly low prevalence settings [42]. This may be particularly relevant in the Indian context, as part of the slides examined for malaria are not obtained from patients seeking medical care but from active surveillance (see Methods).

In our model, the reservoir provided by infectious individuals that lack severe symptoms plays a dynamical role primarily at seasonal scales in the decay and trough of epidemics [29] rather than at interannual ones, with the interannual signal in the data largely captured by the variability of the rainfall forcing. Future work with this more realistic model should consider independent measurements of specific epidemiological parameters, as well as the age distribution of clinical and non-clinical infections, to constrain the dimensionality of the search. This would allow a better understanding of the possible mechanisms and temporal scales of a reservoir in transmission, given that class 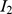 plays two different roles in this model that are difficult to separate: it provides a reservoir for infection at low levels and for longer times than the symptomatic class 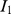, while also keeping individuals protected from clinical disease. Our results suggest that this protection might be long lasting, once acquired, requiring only low levels of re-infection (with a low value of the coefficient 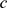). This may reflect a low (antigenic) diversity of *P. falciparum* in these regions of low transmission intensity. In its current formulation, this kind of long-lasting protection is obtained by making 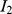 long lasting with only a very small fraction 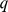 of individuals in this class contributing to the force of infection. A more realistic formulation, currently under investigation, would require and extension of the model that incorporates more continuous levels of susceptibility and infection without increasing model complexity.

Alternatives to our likelihood-based approach for inference on nonlinear dynamic systems include spectral matching [43], gradient matching [44] and Bayesian methodology [45], [46]. Our choice of likelihood-based methods was influenced by their statistical efficiency (even in the face of poor estimability of some parameters [47]), the availability of computationally efficient numerical algorithms [21], and the lack of scientifically supported prior distributions for a Bayesian analysis. However, alternative methods could lead to complementary perspectives on data analysis. All statistical methods can be expected to give valid conclusions only when the model under consideration is adequate for the investigation at hand. By comparing a range of models, including empirical statistical models, we can be confident that our mechanistic models have a reasonable level of statistical fit, even once they are penalized for additional model complexity. We cannot, however, rule out the possible existence of superior models leading potentially to differing conclusions. Indeed, we hope and expect that future work will refine the models that we have presented. We anticipate the development of a body of research investigating and explaining fluctuations in epidemic malaria, based on confronting dynamic models to population-level time series data.

Finally, our results indicate the feasibility of forecasting malaria epidemics in these desert and semi-arid regions of India based on climate variability. Our epidemiological models including rainfall exhibited high prediction skill for seasonal cases as a function of monsoonal rainfall. This skill is comparable to, and even higher than, that of a purely statistical model that incorporates a threshold and increasing uncertainty with rainfall. Thus, these aspects of the nonlinear response to climate variability, and not the nonlinear dynamics of the disease itself, appear key to variation and prediction of total epidemic size in these regions. However, disease dynamics appear useful to predict the time course of the epidemic curve, that is, the rise and fall of the individual outbreaks following the monsoons. Future investigations should consider other districts to encompass a larger geographic area, as well as the effects of control efforts, socio-economic conditions, and related land-use patterns including irrigation, to tackle the remaining unpredictability in the size of large epidemics. Other aspects of rainfall variability, in particular those pertaining to the monsoon season, should be examined to consider not just short-term prediction but possible implications of changes in the intensity and frequency of extremes with climate change in India [48]. Longer lead times for prediction should also be explored based on climate dynamics and global scale drivers of rainfall.

## Methods

The malaria data consists of monthly clinical cases from positive slides of *Plasmodium falciparum* from 1987 to 2007 in Kutch and from 1985 to 2005 in Barmer, two large semi-arid and desert districts of North-West India in the states of Gujarat and Rajasthan respectively (Figure 1A and Figure S3A; see also map in Figure S1). The epidemiological data reported by the health system are based on two mechanisms: (a) active surveillance on a fortnight basis: collection of blood slides from fever patients by house to house visits by a health worker and examination of these slides for malaria parasites at the Primary/Community Health Center of that area; (b) passive surveillance: examination of blood slides from fever patients reporting directly to the Primary/Community Health Center. Both types of data are pooled and analyzed for each village. For this study, epidemiological data were collected from the office of the District Malaria Officer.

Monthly accumulated rainfall for 20 years of data was obtained from local weather stations (Figure S2). Monthly rainfall data were supplied by the Indian Meteorological Department, Pune (India). For Kutch rainfall was recorded at Bhuj located at 23 15′ N, 69 49′ E and for Barmer at a station located at 25 45′ N, 71 25′ E. Time series for total population size were obtained via interpolation from census data available every 10 years, i.e., in 1990 and 2000.

For our malaria transmission models, we do not rely on the well-known Ross-Macdonald formulation and its extensions [49], [50] because they assume that the human and mosquito populations are constant, and track the respective fractions infected. In epidemic regions, mosquito abundances are highly dynamic. Malaria further differs from the well-known models of childhood diseases such as measles that have been studied extensively on the parameter inference front [45], [51], in two ways: transmission by a vector and more complex, waning patterns of immunity that have been represented with different structures of the human population (e.g. [25], [26]). An innovative feature of our model is the inclusion of a parsimonious representation of the vector dynamics motivated by our inference goals. The stage *κ* represents the latent force of infection or latent per-capita rate of infection from an infected to a susceptible human. This is not the realized force of infection because a mosquito that acquires infection by biting an infective or infectious human cannot immediately transmit the disease through a second bite. The *Plasmodium* parasite needs to first complete its incubation period, and to do so before the vector dies. To account for the development of the *Plasmodium* parasite among surviving mosquitoes, we introduce a second variable, *λ*, representing the current force of infection and consisting of the latent infection lagged by a distributed delay. We specifically consider Gamma-distributed transitions [52], [53] for the latent period of the force of infection, these are more flexible than the more standard exponential and better suited to developmental times that give rise to a (temperature-dependent) lower bound. This parsimonious representation of the vector can be mapped explicitly to, and derived from, the well-known parameters and treatment of mosquitoes in standard malaria models, by rewriting these models as non-autonomous with mosquito abundance as the forcing (see Text S1).

By representing mosquito dynamics implicitly through a model for the force of infection of humans, we avoid explicit consideration of mosquito abundance, survival and behavior. In the absence of mosquito data, we limit our inclusion of vector dynamics to the aspect that is most directly relevant to the human disease. We consider that the main stochasticity in this system arises from variations in vector abundance and behavior, and model this by forcing the rate of change of *κ* with three different sources of exogenous variability, namely seasonality, climate covariates (here rainfall), and random environmental noise (multiplicative Gamma noise) (see Text S1 for details). The form of the climate covariate in this forcing is given by 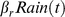 with 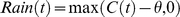, a function of the accumulated rainfall 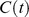 over the past 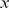 months. Accumulated rainfall is given by 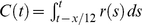 where 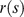 is a spline interpolation of the discretely measured monthly rainfall. Here, 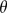 is a threshold for accumulated rainfall, and 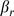 is a constant coefficient. From preliminary investigations based on likelihood profiles, we selected 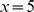 months and 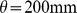 for Kutch and Barmer. Seasonality is modeled nonparametrically.

For the human component, we adopt first the well-established practice of subdividing the population into the following distinct classes: 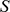, susceptible to infection (and disease); 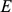, exposed (i.e., carrying *Plasmodium* parasites which have not yet matured into gametocytes); 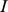, infected and gametocytemic (infectious); and 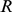, recovered and protected from all but asymptomatic and negligibly gametocytemic reinfections (Figure 2A). The total population size 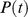 is supposed known by interpolation from census, and the birth rate into the susceptible class is set to ensure that 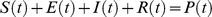. We refer to this model hereafter as VSEIRS (for the vector and population classes respectively). To examine the robustness of our results, we consider then a more complex representation of immunity that differentiates between two classes of infected individuals, and in so doing, adds the possibility of clinical immunity (Figure 2B, 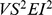). This formulation follows that of [25] and differentiates between susceptibility to disease and infection [24]. It is one of several possible representations that follow from the pioneer work of Dietz *et al.* (1974) on malaria models that recognize different levels and roles of immunity. We incorporate here two classes of infected individuals, corresponding to two levels of infection, for clinical and asymptomatic cases, respectively. The latter retain the ability to transmit to mosquitoes but at a reduced rate. Two classes of susceptibles allow us to differentiate individuals lacking protection to clinical disease from those protected from disease but retaining susceptibility to (asymptomatic) infection. Thus this model incorporates clinical immunity (classes 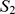 and 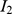) but does not allow for the development of full immunity to infection; instead, individuals can be re-infected and maintain in this way their protection to disease. This is meant to represent in a simple way that immunity to disease is acquired by repeated exposure [54], [55], and its maintenance depends on repeated re-infection [26], [50], [56]–[58]. The model effectively introduces two pathways, and therefore, two different temporal scales, for the acquisition and loss of immunity: a first pathway between fully susceptible individuals and severe infection and back, which allows for repeated symptomatic infections and the resulting acquisition of protection from disease; a second pathway through less severe infections, which potentially allows for a longer lasting removal from the pool of individuals susceptible to disease. Sustaining clinical immunity (by keeping individuals in this pathway) would require very different levels of re-infection (from 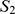 to 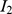) in different regions depending on the parasite's (antigenic) diversity and therefore, on transmission intensity. In epidemic regions, where such diversity is presumably low, the rate of repeated re-infections would also be low but individuals may be nevertheless effectively protected from clinical disease for a long time once they have transitioned to asymptomatic infection because they have been exposed to much of the existing diversity. The corresponding system of stochastic differential equations and details on the seasonal and stochastic forcing are given in Text S1.

To complete the model, we need to specify the relationship between the continuous-time dynamic system 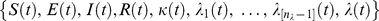 and the data on monthly reported malaria cases 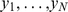 at the discrete set of observation times 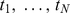. We assume that, on average, a fraction 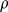 of the people moving from class 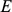 to class 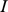 are detected by the surveillance system. Specifically, we model 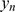 conditional on the history of the dynamic system as
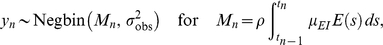
(1)where 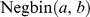 is the negative binomial distribution with mean 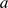 and variance 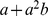. The negative binomial distribution provides a model for count data that includes the possibility of overdispersion relative to Poisson or binomial models, and permits both under-reporting and over-reporting. To estimate parameters but also to compare among different models representing different hypotheses on the origin of the interannual variation of malaria epidemics, we used a recently developed likelihood-based inference technique for stochastic differential equations based on iterating filtering, a sequential Monte Carlo optimization method [21], [22], [51]. The method allows the comparison of different mechanistic models that include stochasticity, non-linearity, and non-observed states (see Text S1). The algorithm is briefly explained and summarized in Text S1. We implemented iterated filtering via the mif function of the R package pomp [59], which carries out the algorithm detailed in the supplement to [22].

To investigate the information in the data about specific parameters, in the absence of constraints on other parameters, we used profile likelihood methods. The confidence intervals resulting from these profiles are based on likelihood ratio tests. Thus, our confidence intervals enjoy the properties of likelihood ratio tests and are in particular robust to weak identifiability of other parameters [47]. Of course, inasmuch as the parameter itself is weakly identified, its profile will be flat and its confidence interval wide.

To investigate forecasting, we focus on the task of predicting the total malaria incidence in the peak transmission months of September through December based on information available at the end of August. We challenge the different models to predict the reported incidence rather than the actual number of cases: the former measures the burden on the public health services and the latter is an unobserved quantity which is linked to the reported cases through an unknown reporting rate. We compare mechanistic models with and without rainfall, a linear prediction based on accumulated rainfall during the May through August monsoon season, and a nonlinear mixture model where the chance of the epidemic component of the mixture depends on accumulated rainfall [60]. The main idea of the mixture model is to capture the threshold response of cases as a function of an environmental covariate (here, rainfall), and allow for different means and variances as a function of this covariate. We constructed a mixture model motivated by [60] in which the Poisson mixture components of [60] were replaced with a negative binomial distribution (see details in Text S1). This overdispersed distribution is better suited to the large variance of the data (Figure 1C and Figure S3C), given the limitation that the variance equals the mean imposed by a Poisson distribution. The predictive ability of the models was evaluated retrospectively in two different ways: first, prediction skill was measured by comparing for each season the error of the model's prediction to that of a trivial and uninformative model that simply predicts the mean total cases, with both errors normalized by a variance (see Text S1). This tells us how much better is our ability to predict than that of trivially using the mean. A value close to one indicates high prediction skill, whereas a value close to zero or even negative indicates poor skill. The second approach is based on likelihoods and evaluates how likely the observed total number of cases is for a given season, given forecasts from the different models. This approach is illustrated in detail in Text S1 and Figure S4.

To examine the dominant temporal scales present in the observed and simulated time series, we used wavelet analysis (e.g. [27], [28]). The wavelet spectrum differs from its predecessor, the Fourier power spectrum, in that it describes the distribution of the variance in the data not just as a function of the different frequencies but also as a function of their localization in time. This is achieved by decomposing the signal with a family of functions (wavelets) whose support is local, and differs in this way from the sines and cosines of Fourier analysis. The local nature of the wavelet power spectrum makes it better suited to characterize patterns of variability whose dominant periods change over time. See Cazelles et al. [27] for a detailed explanation of wavelet analysis in the context of population dynamics, and [28] for an application to epidemiology.

## Supporting Information

Figure S1 Studied districts in Northwest India.
(2.80 MB TIF)

Figure S2 Seasonality of rainfall and reported cases for Kutch, 1987–2007. (A) Superimposed monthly rainfall; (B) Superimposed monthly cases. Some extreme years are highlighted: 1988 in black, 1989 in red, 1992 in green, 1994 in orange, and 2003 in blue.
(0.72 MB TIF)

Figure S3 Malaria cases and rainfall for Barmer (c.f. Figure 1 for Kutch). (A) Monthly *P. falciparum* malaria reported cases (red) and monthly rainfall from local stations (black) for Barmer. (B) The same rainfall data is shown here with the monthly malaria cases in a logarithmic scale, which emphasizes the patterns of the outbreaks other than the extreme event of 1994–1995. (C) Correlation between accumulated rainfall in the previous months to the months of the cases. A maximum is observed when rainfall is accumulated for 4 to 6 months (D) Correlation between accumulated cases in the previous five months to the month of the cases. A threshold non-linear response of cases to accumulated rainfall can be noticed, with a threshold of around 200mm.
(1.08 MB TIF)

Figure S4 Prediction likelihood density for the year 2006 and the VSEIRS model with rainfall. The histogram is produced from one thousand simulations. Red line shows a kernel density estimate using a bandwith of 0.45 and a gaussian kernel. Given a value of observed cases (on x axis), we can use this curve to determine its likelihood when predicting with a given model (on the y axis).
(1.47 MB TIF)

Figure S5 Reported monthly malaria cases (red) and simulations for Barmer (1985–2005) in a logarithmic scale. Black lines show the median of ten thousand simulations; the shadowed regions correspond to the range between the 10% and 90% percentiles of the simulations. (A) VSEIRS model with rainfall; (B) VSEIRS model without rainfall.
(1.08 MB TIF)

Figure S6 Profile likelihood plot of the duration of immunity 1/μ_RS_. The upper panel corresponds to Kutch; the lower one, to Barmer. Red and blue represent the models with and without rainfall respectively. The dashed vertical lines construct approximate 95% confidence intervals. (A) For Kutch, the duration of immunity is estimated to fall in the interval (0.02,1) or (4.10,5.86) years for the VSEIRS model with rainfall, and (0,23.71) years for the VSEIRS model without rainfall. (B) For Barmer, the duration of immunity is estimated to lie in the interval (0.43,6.76) years for the VSEIRS model with rainfall and is not estimable for the VSEIRS model without rainfall (i.e., the profile is effectively flat).
(1.08 MB TIF)

Figure S7 Profile likelihood plot of reporting rate (ρ) for Kutch (upper panel) and Barmer (lower panel), for the VSEIRS model with rainfall. The dashed vertical lines construct approximate 95% confidence interval. (A) The estimated reporting rate is between 0.3 and 1.9 percent of new infections for Kutch. (B) The estimated reporting rate is between 0.7 and 4.5 percent of new infections for Barmer.
(1.08 MB TIF)

Figure S8 Profile likelihood plot for the mean duration of the delay between the latent and the current force of infection, for Kutch (upper panel) and Barmer (lower panel), for the VSEIRS model with rainfall. The dashed vertical lines construct approximate 95% confidence interval. (A) The estimated delay τ is between 4 and 30 days for for Kutch. (B) The estimated delay τ is between 16 and 39 days for Barmer.
(1.08 MB TIF)

Figure S9 Intermediate model: Reported monthly malaria cases (red) and simulations. Black lines show the median of ten thousand simulations; the shadowed regions correspond to the range between the 10% and 90% percentiles of the simulations. (A) Kutch; (B) Barmer.
(1.08 MB TIF)

Figure S10 Profile likelihood plot for the mean duration of the delay between the latent and the current force of infection, for Kutch for the VS^2^EI^2^ model with rainfall. The dashed vertical lines construct approximate 95% confidence interval. The estimated delay, τ, is between 6.2 and 28.4 days.
(1.47 MB TIF)

Figure S11 Hindcast predictions for the time course of epidemics for Kutch. The malaria data are shown in red. Superimposed on these observations, we show the mean of predicted cases from one to four months ahead obtained by simulating the VS^2^EI^2^ model from (1) the end of August (solid dots in blue) and (2) the end of December (solid dots in green). Shadowed regions in respective colors correspond to the standard deviation from a set of 5000 predicted values. (A) VS^2^EI^2^ model with rainfall; (B) VS^2^EI^2^ model without rainfall. (see further details in the caption of Figure 4).
(1.47 MB TIF)

Figure S12 Wavelet power spectra for simulations, malaria cases and rainfall in Kutch. On the left panels, the y-axis corresponds to the period in years and the x axis, to time. The colors code for the value of the power for a given time and period, from low values in dark blue, to high values, in dark red. The black continuous line gives the boundary within which these values are not influenced by edge effects and are therefore considered reliable, i.e. the cone of influence. The respective right panel represents the global power spectrum (obtained by averaging the wavelet spectrum over time), and is therefore, comparable to a Fourier spectrum. The white lines track the local maxima of the power in the wavelet spectrum. The discontinuous black line corresponds to the 5% significance level (obtained by a bootstrap significance test detailed in Cazelles et. al. 2007): the areas within this line indicate significant variability at the corresponding periods and times. A) Simulation from the MLE of the VS^2^EI^2^ model with rainfall. B) *P. falciparum* malaria cases. C) Rainfall.
(1.23 MB TIF)

Figure S13 Wavelet power spectra for simulations from VSEIRS models. See caption of Figure S12 for details. A) Simulation from the maximum likelihood solution (MLE) of the SEIRS model without rainfall. B) Simulation from the SEIRS model with rainfall with the MLE in the short immunity region (immunity = 1.2 months; log-likelihood = −1265.0). C) Simulation from the SEIRS model with rainfall with the MLE in the long immunity region (immunity = 5 years; log-likelihood = −1266.8).
(1.10 MB TIF)

Table S1 Table of log-likelihood *l* and AIC of the fitted models for Kutch and Barmer. In the table “p” denotes the number of parameters for each model. AIC is computed by the formula AIC = −2 *l* + 2p. The SARIMA model was fitted to the data on the log scale (see the supplement of He et. al. 2010 for a detailed description of this procedure).
(0.03 MB PDF)

Table S2 List of symbols for the malaria model. Fixed parameters are β = 1 yr^−1^, n_λ_ = 1, Δ = 1 day and 1/δ = 50 yr.
(0.03 MB PDF)

Table S3 Estimated model parameters for Kutch. Corresponding units and parameter descriptions are given in Table S2. The columns marked VSEIRS and VS^2^EI^2^ correspond to maximum likelihood point estimates for each type of model, with and without including rainfall. The last two columns give the lower and upper bounds for approximate 95% confidence intervals for the VS^2^EI^2^ model with rainfall, derived from profile likelihood computations as shown in Figures S7 and S8; values of 0, 1 and ∞ correspond to confidence intervals extending to the boundary of the parameter space.
(0.05 MB PDF)

Table S4 Point estimates for estimated parameters of the VSEIRS model with and without rainfall for Barmer district. Corresponding description and units are given in Table S2.
(0.03 MB PDF)

Text S1 Supporting Information online.
(0.12 MB PDF)
