# Supplementary figures and images for: Forcing Versus Feedback: Epidemic Malaria and Monsoon Rains in Northwest India
Source: PLoS Comput Biol. 2010 Sep 2;6(9):e1000898. doi: 10.1371/journal.pcbi.1000898 (PMC2932675; doi:10.1371/journal.pcbi.1000898)

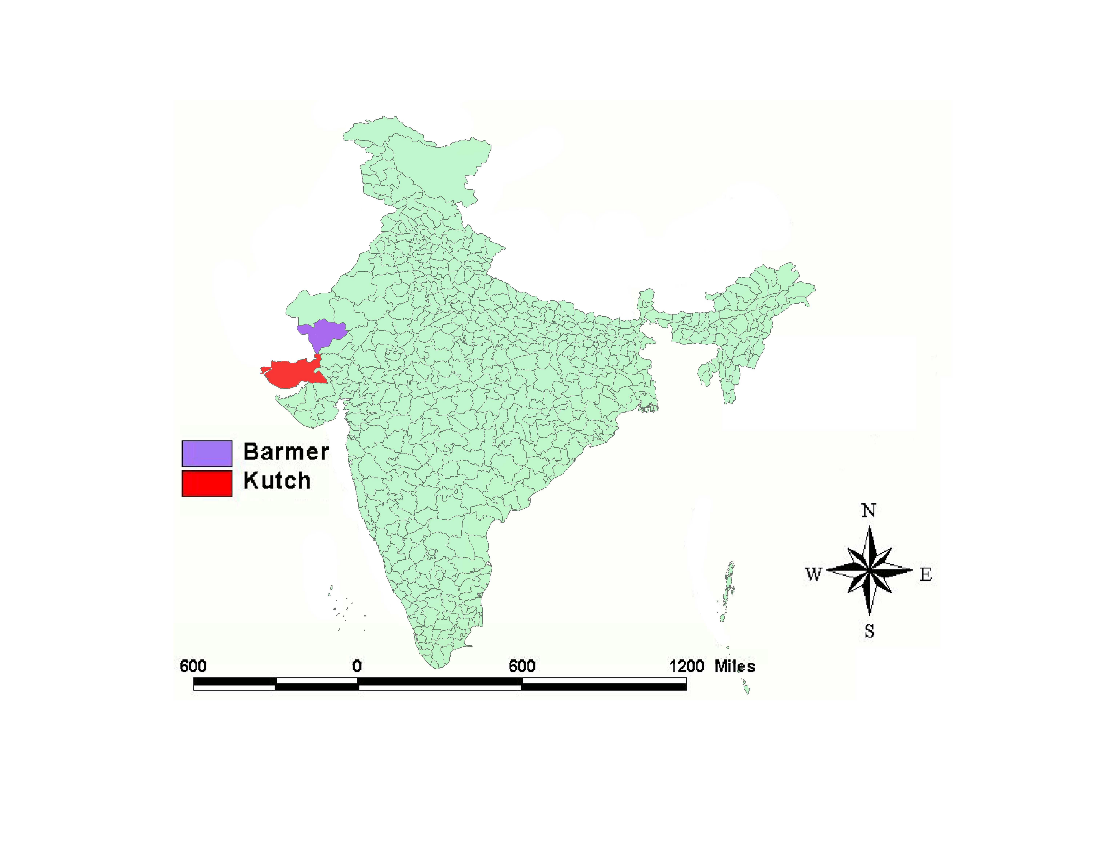

Supplement: Figure S1 — Studied districts in Northwest India. (2.80 MB TIF) [file pcbi.1000898.s001.tif]

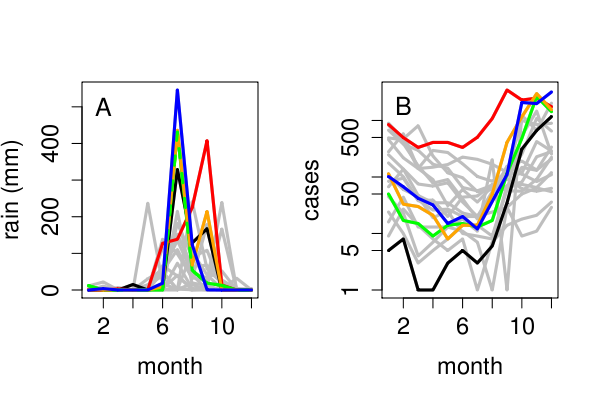

Supplement: Figure S2 — Seasonality of rainfall and reported cases for Kutch, 1987–2007. (A) Superimposed monthly rainfall; (B) Superimposed monthly cases. Some extreme years are highlighted: 1988 in black, 1989 in red, 1992 in green, 1994 in orange, and 2003 in blue. (0.72 MB TIF) [file pcbi.1000898.s002.tif]

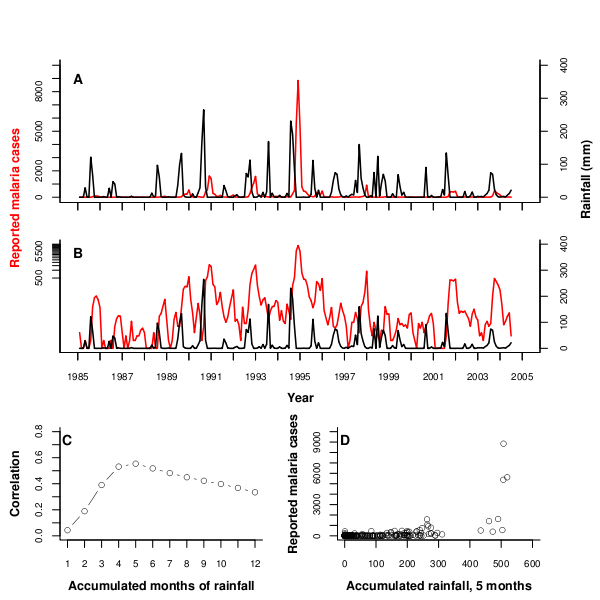

Supplement: Figure S3 — Malaria cases and rainfall for Barmer (c.f. Figure 1 for Kutch). (A) Monthly P. falciparum malaria reported cases (red) and monthly rainfall from local stations (black) for Barmer. (B) The same rainfall data is shown here with the monthly malaria cases in a logarithmic scale, which emphasizes the patterns of the outbreaks other than the extreme event of 1994–1995. (C) Correlation between accumulated rainfall in the previous months to the months of the cases. A maximum is observed when rainfall is accumulated for 4 to 6 months (D) Correlation between accumulated cases in the previous five months to the month of the cases. A threshold non-linear response of cases to accumulated rainfall can be noticed, with a threshold of around 200mm. (1.08 MB TIF) [file pcbi.1000898.s003.tif]

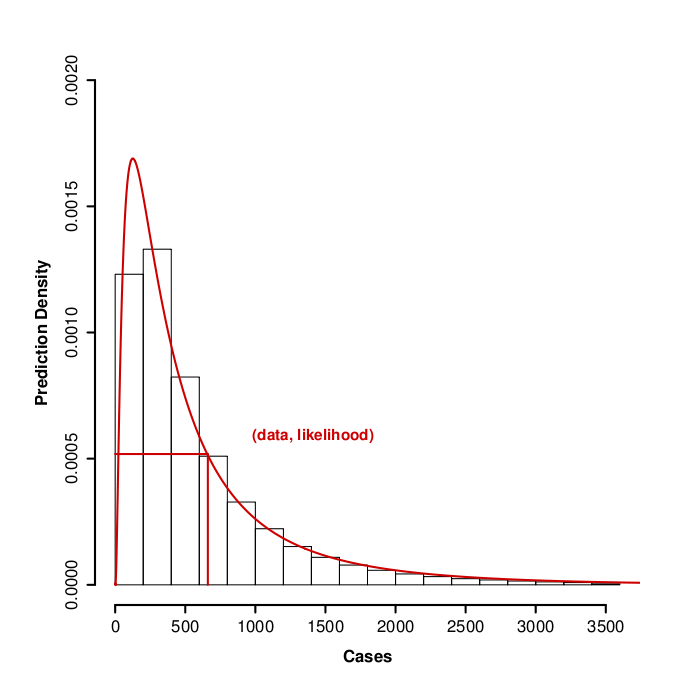

Supplement: Figure S4 — Prediction likelihood density for the year 2006 and the VSEIRS model with rainfall. The histogram is produced from one thousand simulations. Red line shows a kernel density estimate using a bandwith of 0.45 and a gaussian kernel. Given a value of observed cases (on x axis), we can use this curve to determine its likelihood when predicting with a given model (on the y axis). (1.47 MB TIF) [file pcbi.1000898.s004.tif]

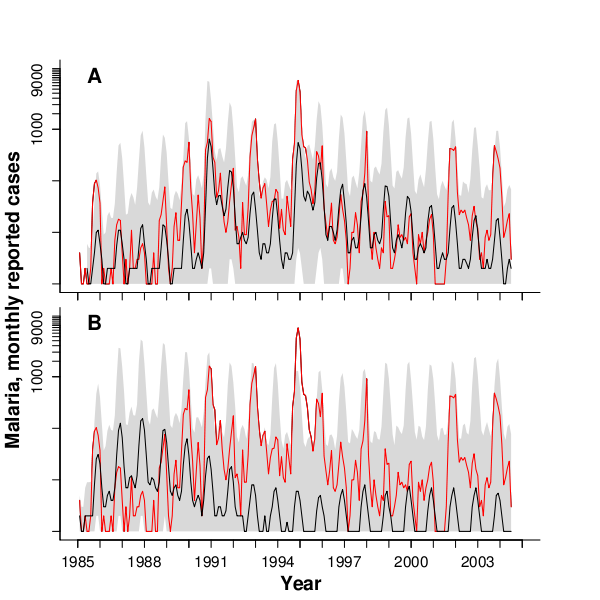

Supplement: Figure S5 — Reported monthly malaria cases (red) and simulations for Barmer (1985–2005) in a logarithmic scale. Black lines show the median of ten thousand simulations; the shadowed regions correspond to the range between the 10% and 90% percentiles of the simulations. (A) VSEIRS model with rainfall; (B) VSEIRS model without rainfall. (1.08 MB TIF) [file pcbi.1000898.s005.tif]

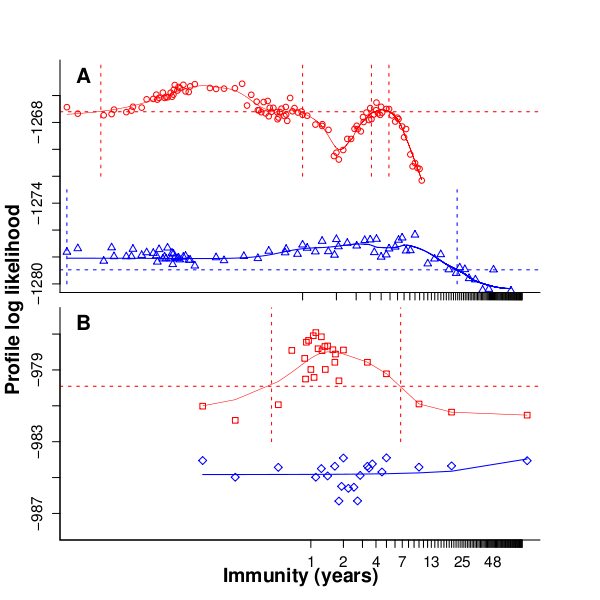

Supplement: Figure S6 — Profile likelihood plot of the duration of immunity 1/μRS. The upper panel corresponds to Kutch; the lower one, to Barmer. Red and blue represent the models with and without rainfall respectively. The dashed vertical lines construct approximate 95% confidence intervals. (A) For Kutch, the duration of immunity is estimated to fall in the interval (0.02,1) or (4.10,5.86) years for the VSEIRS model with rainfall, and (0,23.71) years for the VSEIRS model without rainfall. (B) For Barmer, the duration of immunity is estimated to lie in the interval (0.43,6.76) years for the VSEIRS model with rainfall and is not estimable for the VSEIRS model without rainfall (i.e., the profile is effectively flat). (1.08 MB TIF) [file pcbi.1000898.s006.tif]

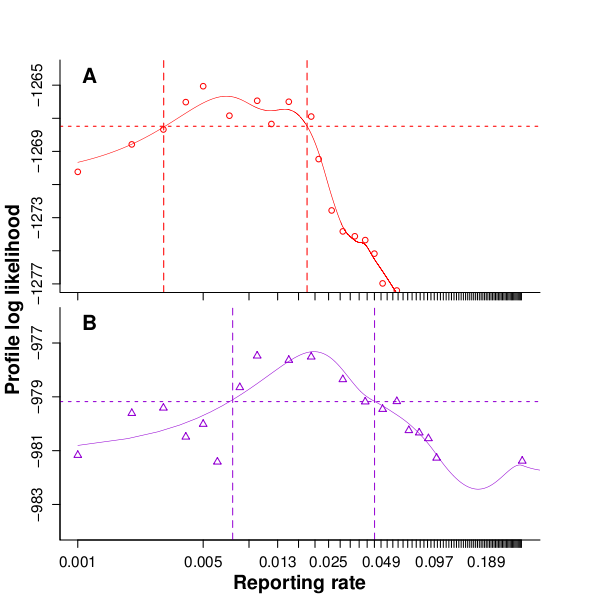

Supplement: Figure S7 — Profile likelihood plot of reporting rate (ρ) for Kutch (upper panel) and Barmer (lower panel), for the VSEIRS model with rainfall. The dashed vertical lines construct approximate 95% confidence interval. (A) The estimated reporting rate is between 0.3 and 1.9 percent of new infections for Kutch. (B) The estimated reporting rate is between 0.7 and 4.5 percent of new infections for Barmer. (1.08 MB TIF) [file pcbi.1000898.s007.tif]

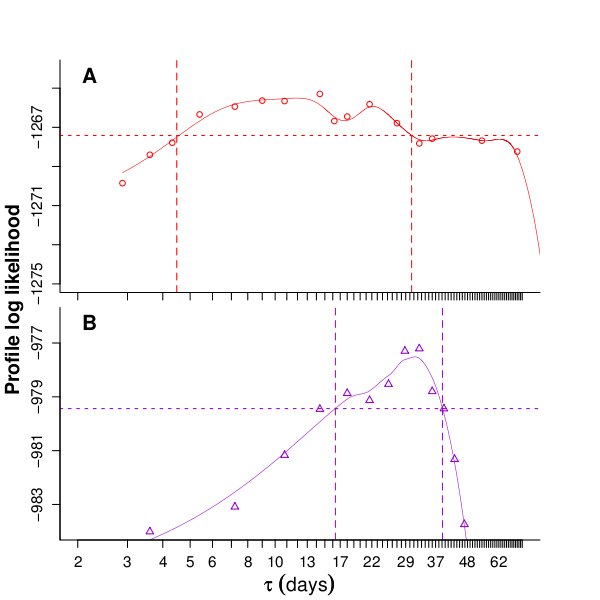

Supplement: Figure S8 — Profile likelihood plot for the mean duration of the delay between the latent and the current force of infection, for Kutch (upper panel) and Barmer (lower panel), for the VSEIRS model with rainfall. The dashed vertical lines construct approximate 95% confidence interval. (A) The estimated delay τ is between 4 and 30 days for for Kutch. (B) The estimated delay τ is between 16 and 39 days for Barmer. (1.08 MB TIF) [file pcbi.1000898.s008.tif]

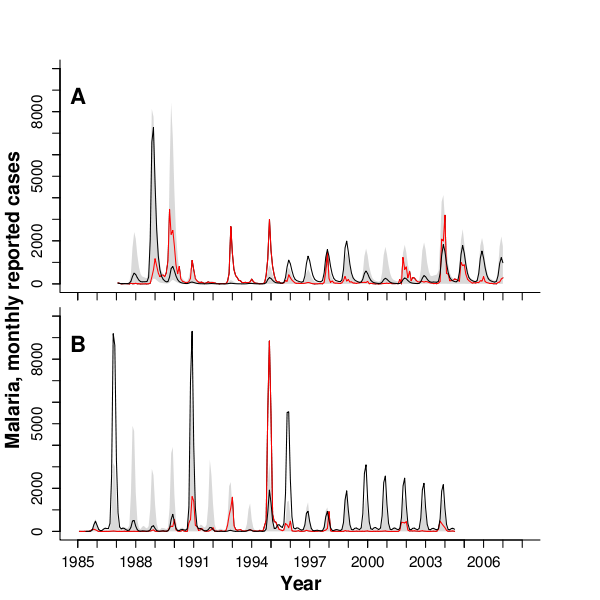

Supplement: Figure S9 — Intermediate model: Reported monthly malaria cases (red) and simulations. Black lines show the median of ten thousand simulations; the shadowed regions correspond to the range between the 10% and 90% percentiles of the simulations. (A) Kutch; (B) Barmer. (1.08 MB TIF) [file pcbi.1000898.s009.tif]

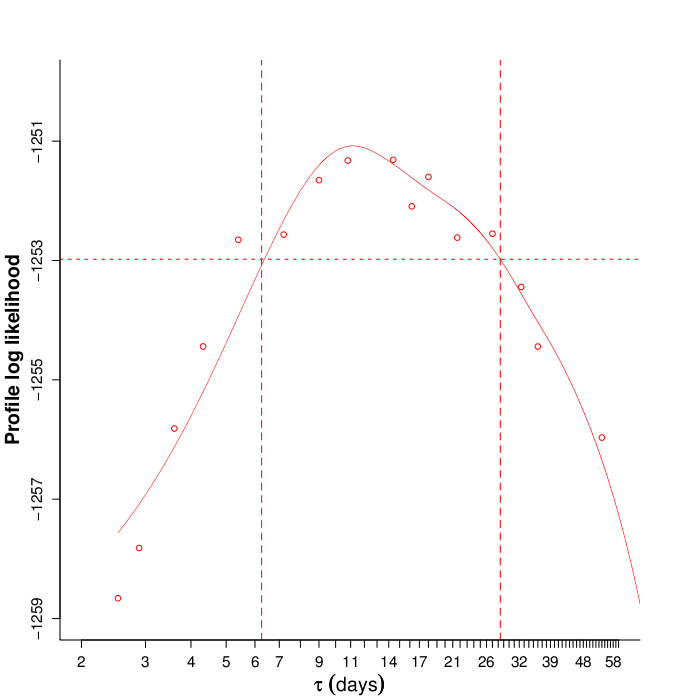

Supplement: Figure S10 — Profile likelihood plot for the mean duration of the delay between the latent and the current force of infection, for Kutch for the VS2EI2 model with rainfall. The dashed vertical lines construct approximate 95% confidence interval. The estimated delay, τ, is between 6.2 and 28.4 days. (1.47 MB TIF) [file pcbi.1000898.s010.tif]

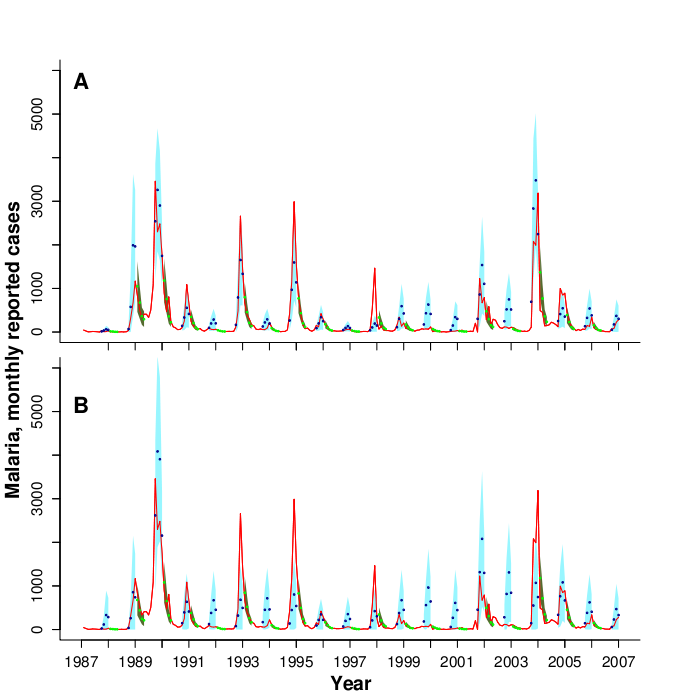

Supplement: Figure S11 — Hindcast predictions for the time course of epidemics for Kutch. The malaria data are shown in red. Superimposed on these observations, we show the mean of predicted cases from one to four months ahead obtained by simulating the VS2EI2 model from (1) the end of August (solid dots in blue) and (2) the end of December (solid dots in green). Shadowed regions in respective colors correspond to the standard deviation from a set of 5000 predicted values. (A) VS2EI2 model with rainfall; (B) VS2EI2 model without rainfall. (see further details in the caption of Figure 4). (1.47 MB TIF) [file pcbi.1000898.s011.tif]

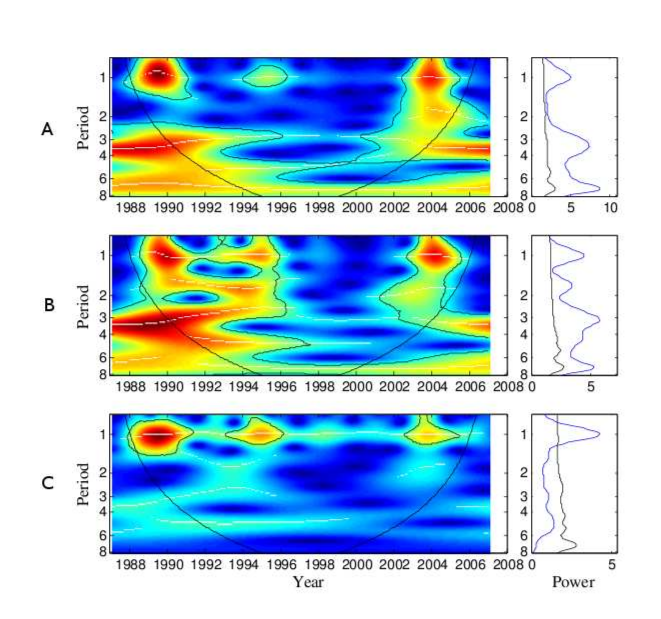

Supplement: Figure S12 — Wavelet power spectra for simulations, malaria cases and rainfall in Kutch. On the left panels, the y-axis corresponds to the period in years and the x axis, to time. The colors code for the value of the power for a given time and period, from low values in dark blue, to high values, in dark red. The black continuous line gives the boundary within which these values are not influenced by edge effects and are therefore considered reliable, i.e. the cone of influence. The respective right panel represents the global power spectrum (obtained by averaging the wavelet spectrum over time), and is therefore, comparable to a Fourier spectrum. The white lines track the local maxima of the power in the wavelet spectrum. The discontinuous black line corresponds to the 5% significance level (obtained by a bootstrap significance test detailed in Cazelles et. al. 2007): the areas within this line indicate significant variability at the corresponding periods and times. A) Simulation from the MLE of the VS2EI2 model with rainfall. B) P. falciparum malaria cases. C) Rainfall. (1.23 MB TIF) [file pcbi.1000898.s012.tif]

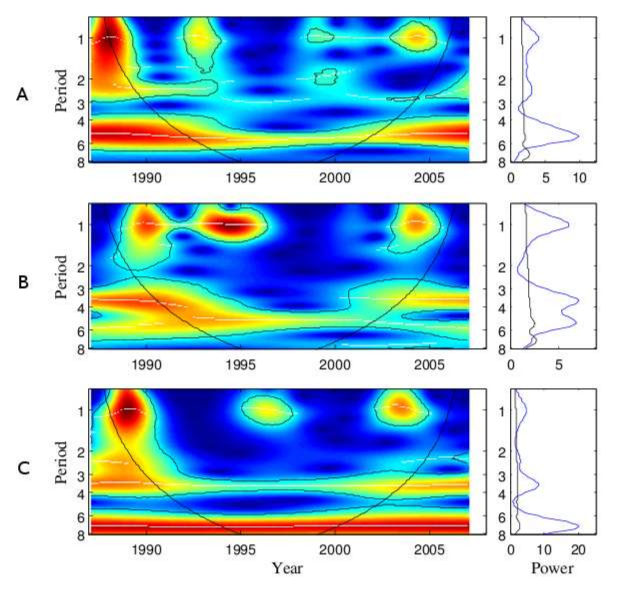

Supplement: Figure S13 — Wavelet power spectra for simulations from VSEIRS models. See caption of Figure S12 for details. A) Simulation from the maximum likelihood solution (MLE) of the SEIRS model without rainfall. B) Simulation from the SEIRS model with rainfall with the MLE in the short immunity region (immunity = 1.2 months; log-likelihood = −1265.0). C) Simulation from the SEIRS model with rainfall with the MLE in the long immunity region (immunity = 5 years; log-likelihood = −1266.8). (1.10 MB TIF) [file pcbi.1000898.s013.tif]
