# Supplementary material for: Forcing Versus Feedback: Epidemic Malaria and Monsoon Rains in Northwest India
Source: PLoS Comput Biol. 2010 Sep 2;6(9):e1000898. doi: 10.1371/journal.pcbi.1000898 (PMC2932675; doi:10.1371/journal.pcbi.1000898)
Supplement: Table S1 — Table of log-likelihood l and AIC of the fitted models for Kutch and Barmer. In the table “p” denotes the number of parameters for each model. AIC is computed by the formula AIC = −2 l + 2p. The SARIMA model was fitted to the data on the log scale (see the supplement of He et. al. 2010 for a detailed description of this procedure). (0.03 MB PDF) [file pcbi.1000898.s014.pdf]

Table S1. Table of log-likelihood (ℓ) and AIC of the fitted models for Kutch and Barmer.

model	p	log-likelihood (ℓ)		AIC	
		Kutch	Barmer	Kutch	Barmer
VSEIRS model without rainfall	19	-1275.0	-984.1	2588.0	2006.2
VSEIRS model with rainfall	20	-1265.0	-978.6	2570.0	1997.2
VS^2EI^2 model without rainfall	24	-1261.1	-975.3	2570.2	1998.6
VS^2EI^2 model with rainfall	25	-1251.0	-970.5	2552.0	1991.0
SARIMA $(1,0,1) \times (1,0,1)_{12}$ without rainfall	6	-1329.0	-983.7	2670.0	1979.4
SARIMA $(1,0,1) \times (1,0,1)_{12}$ with rainfall	7	-1322.6	-977.0	2659.2	1968.0

In the table "p" denotes the number of parameters for each model. AIC is computed by the formula AIC $= -2\ell + 2p$. The SARIMA model was fitted to the data on the log scale (see the supplement of [2] for a detailed description of this procedure).
