# Supplementary material for: Forcing Versus Feedback: Epidemic Malaria and Monsoon Rains in Northwest India
Source: PLoS Comput Biol. 2010 Sep 2;6(9):e1000898. doi: 10.1371/journal.pcbi.1000898 (PMC2932675; doi:10.1371/journal.pcbi.1000898)
Supplement: Table S2 — List of symbols for the malaria model. Fixed parameters are β = 1 yr−1, nλ = 1, Δ = 1 day and 1/δ = 50 yr. (0.03 MB PDF) [file pcbi.1000898.s015.pdf]

Table S2. List of symbols for the malaria model.

symbol	description	unit	estimated? (y/n)
μ_{XY}	per-capita rate of transition from compartment X to Y; X, Y $\in$ {S,E,I,R,I_1,I_2,S_1,S_2}	yr^{-1}	y
β_i	ith spline coefficient	-	y
$\overline{\beta}$	dimensionality constant	yr	n
τ	mean development delay for mosquitoes	yr	y
σ	sd of the process noise	$yr^{1/2}$	y
ρ	reporting fraction	-	y
n_λ	shape parameter for the delay development kernel for mosquitoes	-	n
Δ	time step for stochastic Euler integration	day	n
$1/\delta$	average human life expectancy	yr	n
σ_{obs}	sd of the observation noise	-	y
β_r	coefficient of climate (rainfall) covariate	mm^{-1}	y
X_0	initial fraction of people in compartment X; X $\in$ {S,E,I,R,I_1,I_2,S_1,S_2}	-	y
κ_0, λ_0	Initial values for the latent and current force of infection	-	y
q	Infectivity of quiescent cases relative to full-blown infections	-	y
c	Proportionality constant for superinfection	-	y

Fixed parameters are $\overline{\beta} = 1\text{yr}^-1$, $n_\lambda = 1$, $\Delta = 1$ day and $1/\delta = 50$ yr.
