# Supplementary material for: Forcing Versus Feedback: Epidemic Malaria and Monsoon Rains in Northwest India
Source: PLoS Comput Biol. 2010 Sep 2;6(9):e1000898. doi: 10.1371/journal.pcbi.1000898 (PMC2932675; doi:10.1371/journal.pcbi.1000898)
Supplement: Table S3 — Estimated model parameters for Kutch. Corresponding units and parameter descriptions are given in Table S2. The columns marked VSEIRS and VS2EI2 correspond to maximum likelihood point estimates for each type of model, with and without including rainfall. The last two columns give the lower and upper bounds for approximate 95% confidence intervals for the VS2EI2 model with rainfall, derived from profile likelihood computations as shown in Figures S7 and S8; values of 0, 1 and ∞ correspond to confidence intervals extending to the boundary of the parameter space. (0.05 MB PDF) [file pcbi.1000898.s016.pdf]

Table S3. Estimated model parameters for Kutch.

	Kutch					
	VSEIRS without rain	VS^2EI^2 without rain	VSEIRS with rain	VS^2EI^2 with rain	confidence	interval
μ_{IR}	13.587	–	39.021	–	(–	, –)
μ_{RS}	0.116	–	5.657	–	(–	, –)
$\mu_{S_2S_1}$	–	0.230	–	0.334	(0.067	, 3.270)
μ_{EI}	7.301	7.408	10.480	8.902	(8.885	, 17.277)
$\mu_{I_1I_2}$	–	11.544	–	5.511	(3.218	, ∞)
$\mu_{I_2S_2}$	–	0.004	–	0.035	(0	, 0.073)
$\mu_{I_1S_1}$	–	2.320	–	6.563	(0	, ∞)
β_1	-0.076	-2.469	1.242	1.201	(-4.819	, 4.109)
β_2	1.287	2.001	3.590	2.088	(-0.153	, 6.616)
β_3	4.446	4.227	3.906	3.866	(1.874	, 6.939)
β_4	2.868	2.786	3.747	2.808	(1.092	, 6.042)
β_5	6.709	6.534	5.742	5.996	(4.695	, 9.749)
β_6	6.319	7.080	4.803	5.333	(3.912	, 8.287)
τ	0.025	0.022	0.033	0.030	(0.015	, 0.084)
σ	0.347	0.309	0.225	0.243	(0.162	, 0.259)
ρ	0.022	0.030	0.005	0.015	(0.007	, 0.025)
$q \times 10^4$	–	4.763	–	9.424	(0.100	, 48.102)
σ_{obs}	0.384	0.390	0.390	0.395	(0.365	, 0.445)
β_r	–	–	0.489	0.512	(0.270	, 0.765)
S_0	0.494	–	0.956	–	(–	, –)
$S_{1.0}$	–	0.164	–	0.138	(0.001	, 0.900)
E_0	0.003	0.002	0.014	0.004	(0.003	, 0.009)
I_0	0.011	–	0.002	–	(–	, –)
$I_{1.0}$	–	0.002	–	0.002	(0	, 0.087)
$I_{2.0}$	–	0.067	–	0.080	(0	, 0.754)
R_0	0.505	–	0.038	–	(–	, –)
$S_{2.0}$	–	0.765	–	0.775	(0.276	, 0.900)
$\kappa_0 \times 10$	0.079	0.133	0.189	0.171	(0	, ∞)
$\lambda_0 \times 10$	0.050	0.045	0.058	0.061	(0	, ∞)
c	–	0.004	–	0.010	(0.001	, 0.067)

Corresponding units and parameter descriptions are given in Table S2. The columns marked VSEIRS and VS^2EI^2 correspond to maximum likelihood point estimates for each type of model, with and without including rainfall. The last two columns give the lower and upper bounds for approximate 95% confidence intervals for the VS^2EI^2 model with rainfall, derived from profile likelihood computations as shown in Figures S7 and S8; values of 0, 1 and ∞ correspond to confidence intervals extending to the boundary of the parameter space.
