# Supplementary material for: Forcing Versus Feedback: Epidemic Malaria and Monsoon Rains in Northwest India
Source: PLoS Comput Biol. 2010 Sep 2;6(9):e1000898. doi: 10.1371/journal.pcbi.1000898 (PMC2932675; doi:10.1371/journal.pcbi.1000898)
Supplement: Table S4 — Point estimates for estimated parameters of the VSEIRS model with and without rainfall for Barmer district. Corresponding description and units are given in Table S2. (0.03 MB PDF) [file pcbi.1000898.s017.pdf]

Table S4. Point estimates for estimated parameters of the VSEIRS model with and without rainfall for Barmer district.

	Barmer	
	VSEIRS with rain	VSEIRS without rain
μ_{IR}	39.153	41.164
μ_{RS}	1.009	0.253
μ_{EI}	26.802	24.604
β_1	-3.318	-2.884
β_2	7.477	7.578
β_3	3.057	3.030
β_4	3.296	3.441
β_5	7.453	7.918
β_6	3.525	4.038
τ	0.083	0.079
σ	0.290	0.324
ρ	0.017	0.029
σ_{obs}	0.613	0.598
β_r	0.324	
S_0	0.995	0.950
E_0	0.000	0.000
I_0	0.000	0.000
R_0	0.005	0.051
κ_0	0.000	0.000
λ_0	0.000	0.000

Corresponding description and units are given in Table S2.
