# Supplementary material for: Forcing Versus Feedback: Epidemic Malaria and Monsoon Rains in Northwest India
Source: PLoS Comput Biol. 2010 Sep 2;6(9):e1000898. doi: 10.1371/journal.pcbi.1000898 (PMC2932675; doi:10.1371/journal.pcbi.1000898)
Supplement: Text S1 — Supporting Information online. (0.12 MB PDF) [file pcbi.1000898.s018.pdf]

Supplementary Information

Data

Figure S1 shows the location of the two studied districts in India. Figure S2 shows that the monsoon typically arrives in Kutch around July, with some variation from year to year. Cases subsequently peak in September through December. The size of monsoon rainfall is highly variable, and the highlighted years in Figure S2 show that a relatively large monsoon is associated with a steep increase in the disease burden through the beginning of the winter season.
Figure S3A shows the relationship between rainfall and data for Barmer. A maximum correlation between rainfall and accumulated cases is observed for approximately 4 to 5 months of accumulated rainfall Figure S3B. For the maximum observed correlation, a non-linear relationship between cases and accumulated rainfall is observed Figure S3C, with a threshold around 200mm.

Malaria model

A diagram of our models for malaria transmission were given in Figure 2. For the VSEIRS model (Figure 2 A), the corresponding system of stochastic differential equations is given by:

$$\frac{dS}{dt} = \mu_{BS}P - \mu_{SE}S + \mu_{RS}R - \delta S \quad \text{(S1)}$$

$$\frac{dE}{dt} = \mu_{SE}S - \mu_{EI}E - \delta E \quad \text{(S2)}$$

$$\frac{dI}{dt} = \mu_{EI}E - \mu_{IR}I - \delta I \quad \text{(S3)}$$

$$\frac{dR}{dt} = -\mu_{RS}R + \mu_{IR}I - \delta R \quad \text{(S4)}$$

$$\frac{d\kappa}{dt} = \frac{d\lambda_0}{dt} = n_\lambda \, \frac{1}{\tau} \, (f(t) - \kappa) \quad \text{(S5)}$$

$$\frac{d\lambda_i}{dt} = n_\lambda \, \frac{1}{\tau} \, (\lambda_{[i-1]} - \lambda_{[i]}) \qquad \text{for } i = 1, \ldots, n_\lambda - 1 \quad \text{(S6)}$$

$$\frac{d\lambda}{dt} = \frac{d\lambda_{n_\lambda}}{dt} = n_\lambda \, \frac{1}{\tau} \, (\lambda_{[n_\lambda - 1]} - \lambda) \quad \text{(S7)}$$

with

$$f(t) = \left[\frac{I(t)}{P(t)} \exp\left\{ \sum_{i=1}^{n_s} \beta_i s_i(t) + \beta_r R(t) \right\} \frac{d\Gamma}{dt} \right] \overline{\beta}. \quad \text{(S8)}$$

The total population size $P(t)$ is supposed known by interpolation from census, and the birth rate $\mu_{BS}(t)$ is set to ensure that $S(t) + E(t) + I(t) + R(t) = P(t)$. After experimenting with different choices of n_λ, we fixed $n_\lambda = 1$. As described in the main text, the rate of change of the latent force of infection κ is driven by an exogenous forcing, denoted by $f(t)$ and including three sources of variability that influence the vector's abundance and behavior, namely seasonality, climate covariates (here, rainfall), and random noise. Seasonality is modeled nonparametrically through the coefficients $\{\beta_i\}$ of a periodic cubic B-spline basis $\{s_i(t), i = 1, \ldots, n_s\}$ constructed using n_s evenly spaced knots. The results we present all use $n_s = 6$; we also tried $n_s = 12$ and the results were suggestive of a more clearly identified second transmission peak in spring, however the small improvement in fit did not give statistical support for the additional model complexity. Rainfall forcing is represented by $\beta_r R(t)$ as described in the text. Environmental noise is included as multiplicative Gamma noise [1,2]. $\Gamma(t)$ in our model equations denotes a Gamma process representing integrated noise with intensity σ^2 [1,2]. This is defined as a process with stationary independent increments such that $\Gamma(t) - \Gamma(s) \sim \text{Gamma}\big([t-s]/\sigma^2, \sigma^2\big)$ where $\text{Gamma}(a, b)$

is the Gamma distribution with mean ab and variance ab^2. The rationale behind choosing a Gamma noise is that of keeping the term $f(t)$ positive at all times; because a Gamma process is increasing, its derivative is non-negative at all times. This derivative does not exist in the usual sense, but can be given formal meaning in the context of stochastic differential equations (SDEs) in the same way that the non-existent derivative of Brownian motion is formally used to define Gaussian noise. Stochastic Euler methods are applicable for the numerical solution of SDEs driven by Levy noise [3, 4], in a comparable way to the more widely used Gaussian noise [5]. For the continuous-time process in (S1–S8), all the states are necessarily non-negative. When discretizing to give an Euler solution with time step Δ, this property could be violated. However, with $\Delta = 1$ day, such potential numerical issues did not cause problems in our fitted models.

Similarly we can define the system of stochastic differential equations for the VS^2EI^2 (Figure 2 B) model as:

$$\frac{dS_1}{dt} = \mu_{BS_1}P - \mu_{S_1E}S_1 + \mu_{I_1S_1}I_1 + \mu_{S_2S_1}S_2 - \delta S_1 \tag{S9}$$

$$\frac{dE}{dt} = \mu_{S_1E}S_1 - \mu_{EI_1}E - \delta E \tag{S10}$$

$$\frac{dI_1}{dt} = \mu_{EI_1}E - \mu_{I_1S_1}I_1 - \mu_{I_1I_2}I_1 - \delta I_1 \tag{S11}$$

$$\frac{dI_2}{dt} = \mu_{I_1I_2}I + \mu_{S_2I_2}S_2 - \mu_{I_2S_2}I_2 - \delta I_2 \tag{S12}$$

$$\frac{dS_2}{dt} = \mu_{I_2S_2}I_2 - \mu_{S_2S_1}S_2 - \mu_{S_2I_2}S_2 - \delta S_2 \tag{S13}$$

$$\frac{d\kappa}{dt} = \frac{d\lambda_0}{dt} = n_\lambda \frac{1}{\tau}(f(t) - \kappa) \tag{S14}$$

$$\frac{d\lambda_i}{dt} = n_\lambda \frac{1}{\tau}(\lambda_{[i-1]} - \lambda_{[i]}) \qquad \text{for } i = 1, \ldots, n_\lambda - 1 \tag{S15}$$

$$\frac{d\lambda}{dt} = \frac{d\lambda_{n_\lambda}}{dt} = n_\lambda \frac{1}{\tau}(\lambda_{[n_\lambda-1]} - \lambda) \tag{S16}$$

Where in this case the exogenous forcing depends not only on the infected but also on the mildly infected humans through a q constant that represents the infectivity of quiescent cases relative to full-blown infections:

$$f(t) = \left[\frac{I_1(t) + qI_2(t)}{P(t)} \exp\left\{\sum_{i=1}^{n_s} \beta_i s_i(t) + \beta_r R(t)\right\} \frac{d\Gamma}{dt}\right]\overline{\beta}. \tag{S17}$$

In addition we suppose that the rate of superinfection is proportional to the rate of infection $\mu_{S_2I_2} = c\mu_{S_1E}$ with some constant of proportionality $0 \le c \le 1$.

Treatment of vector dynamics

One way to understand our implicit treatment of vector dynamics, through the latent and current force of infection, is to consider for a moment a more complex model that coupled explicitly the dynamics of the vector population (e.g. larvae and adults mosquitoes, the latter subdivided in turn into exposed, infected and uninfected). We could then write the force of infection as follows:

$$f(t) = ba^2c\, M \int_{t_0}^{t} \frac{I(u)}{P}\, x(u)\, p(t-u)\, du$$

where $x(u)$ is the fraction of uninfected mosquitoes at time u, and M is the total number of mosquitoes. Uninfected mosquitoes become infected with malaria with a probability c when they bite (at a rate a) a

human present in the infected class. Finally, the development-mortality kernel $p(.)$ gives the probability that a mosquito, which acquired the parasite at time u, is still alive and carries a fully developed parasite at time t [6].

In our model, we do not represent the coupling to explicit equations for the dynamics of the vector but consider instead that M (or x) are given as an external forcing. Effectively, a coupled model can be rewritten as a non-autonomous model in which the abundance of the (uninfected) vectors provides a temporal forcing of the dynamics in the human host through the force of infection. Thus, we do not follow M or x explicitly, but consider instead that the variability of x includes seasonality, climate covariates, and random environmental noise. In the above equation for $f(t)$, we can view the expression within the integral as a 'latent' force of infection. The consideration of Gamma distributed transitions between the latent and current force of infection replaces the integral and the development-mortality kernel. In practice, we implement this distribution with a series of compartments between κ and λ following [7, 8].

Fitting the malaria model by maximum likelihood

Fitting partially observed nonlinear stochastic dynamic models to data is a methodological challenge. We estimated parameters with a recently developed method, iterated filtering, that allows the likelihood-based comparison of models of disease transmission. This methodology has a *plug-and-play* property [1,2], meaning that one needs only to numerically simulate the differential equations determining the model (more technically, one does not require explicit evaluation of the state transition densities). This enables comparison among a wide class of models hitherto considered impossible. An overview of an iterated filtering procedure, which converges to the maximum of the likelihood function [9, 10], is presented in Algorithm 1. The computationally challenging step is an application of widely used sequential Monte Carlo techniques [11, 12], described in Algorithm 2. The method consists of two loops, with the external loop essentially iterating an internal, 'filtering' loop, and in so doing generating a new, improved estimate of the parameter values at each iteration. The 'filtering' loop implements a selection process for a large number of 'particles' over time. For each time step, a particle can be seen as a simulation characterized by its own set of parameter values. Particles can survive or die as the result of a resampling process, with probabilities determined by their likelihood given the data. From this selection process over the whole extent of the data, a new estimate of the parameters is generated, and from this estimate, a cloud of new particles is re-initialized in Algorithm 1 using a given noise intensity adjusted by a cooling factor. This noise, as well as the stochasticity in the dynamics of the system itself, provide the variability for the selection process of the particles to act upon.

Input:
initial parameter vector, θ_0
initial noise intensity, σ_0
"cooling" rate, $\alpha < 1$

Procedure (iterated filtering):

For $m = 1, \ldots, M$ {begin iterated filtering loop}

(i) Carry out sequential Monte Carlo filtering (Algorithm 2) on the dynamic model with the unknown parameters performing a random walk starting at θ_{m-1} and having noise intensity σ_{m-1}

(ii) Set θ_m to be a weighted average of the filtered estimates of θ from (i), with weights depending on the uncertainty of these estimates

(iii) Set $\sigma_m = \alpha\sigma_{m-1}$

End For {end iterated filtering loop}

Return:
parameter estimate, θ_M
corresponding likelihood from the last iteration of Algorithm 2

Algorithm 1. Outline of an iterated filtering procedure. Further details can be found in [1, 9, 13, 14].

Forecasting malaria epidemics

We briefly describe below the two statistical models, the way forecasts were obtained with the dynamical models, and the quantitative approaches to comparing the effectiveness of the resulting forecasts.

A linear forecast

Write a_i for the accumulated reported cases during September, October, November and December in year $1987 + i$ for $i = 1, \ldots, n$ with $n = 20$. Write r_i for the corresponding accumulated rainfall during May, June, July and August. A linear regression of a_i on r_i gives rise to a forecast $\hat{a}_i^{(1)}$ which serves as a simple benchmark model. Based on the standard linear-Gaussian regression model, we also obtain a prediction variance $V_i^{(1)}$ which in this case is constant for all i.

A negative binomial mixture forecast

To incorporate a threshold response to rainfall, we modeled a_i conditional on r_i as coming from the mixture

$$a_i = \begin{cases} A_{i1} & \text{with probability } p_i \\ A_{i2} & \text{with probability } 1 - p_i \end{cases}$$

where $p_i = \exp\{b_0 + b_1 r_i\}\big(1 + \exp\{b_0 + b_1 r_i\}\big)$ and $\{A_{ij}\}$ is a collection of independent random variables with $A_{ij} \sim \text{Negbin}(\mu_j, s)$. The parameters $(\mu_1, \mu_2, b_0, b_1, s)$ were estimated by maximum likelihood (the likelihood is given in (S19) below) giving rise to a point prediction and variance estimate of

$$\begin{aligned} \hat{a}_i^{(2)} &= p_i\mu_1 + (1 - p_i)\mu_2, \\ V_i^{(2)} &= p_i[\mu_1 + s\mu_1^2] + (1 - p_i)[\mu_2 + s\mu_2^2] + p_i(1 - p_i)(\mu_1 - \mu_2)^2. \end{aligned}$$

Input:
dynamic model
data $y_1, \dots, y_N$ observed at times $t_1, \dots, t_N$
initial state of the dynamic system at a time $t_0 < t_1$
number of particles, J

Procedure (sequential Monte Carlo):

Initialize particles: set up J copies of the initial state

For $n = 1, \dots, N$ {begin filtering loop}

(i) Move each particle according to the dynamic model from time t_{n-1} to t_n

(ii) Resample the particles J times with probability proportional to their likelihood given the data y_n at time t_n

End For {end filtering loop}

Return:
trajectories of filtered particles
likelihood of data $y_1, \dots, y_N$ derived from (ii)

Algorithm 2. Outline of a sequential Monte Carlo (particle filtering) procedure. Further details can be found in [9, 11–14].

Dynamic model forecasts

The forecasts from the mechanistic stochastic models, with and without rainfall, were calculated as follows. Let $\hat{S}_i$, $\hat{E}_i$, $\hat{I}_i$ and $\hat{R}_i$ be a draw from the conditional distribution of the states $S(t)$, $E(t)$, $I(t)$ and $R(t)$ at the end of August in the ith year, given all information available up to time t. This distribution is called the *filtered distribution*, and draws from this distribution are available as a byproduct of the particle filtering approach used to estimate parameters. An ensemble of Monte Carlo forecasts is then derived by simulating K trajectories, each starting from independent draws from this filtering distribution and subsequently evolving according to the dynamic model over a time interval from the start of September to the end of December. When rainfall appeared as a covariate in the model, these forecasted trajectories were calculated with the observed monthly rainfall up to August (which has a delayed effect on the dynamics) and mean monthly rainfall for September, October, November and December (since these measurements are not available at the time of the forecast). Each trajectory $\big\{\big(S^{[k]}(t), E^{[k]}(t), I^{[k]}(t), R^{[k]}(t)\big), k = 1, \dots, K\big\}$ has corresponding monthly case reports given by equation (1) which are summed to give a forecast $\hat{a}_i^{[k]}$. The ensemble point forecast and its variance are then calculated as

$$\hat{a}_i = \frac{1}{K}\sum_{k=1}^{K} \hat{a}_i^{[k]}; \qquad V_i = \frac{1}{K-1}\sum_{k=1}^{K} (\hat{a}_i^{[k]} - \hat{a}_i)^2. \tag{S18}$$

The results of evaluating (S18) with and without employing rainfall as a covariate are denoted $(\hat{a}_i^{(3)}, V_i^{(3)})$ and $(\hat{a}_i^{(4)}, V_i^{(4)})$ respectively.

Evaluating forecast skill by mean squared error

We test our models for prediction performance over a 4 month period (September to December). During the prediction period, the monthly rainfall covariate is replaced by the respective mean monthly rainfall.

For Kutch we define a skill measure for the models as

$$\text{skill} = 1 - \frac{\sum_{i=1987}^{2007}(y_i - \hat{y}_i)^2 w_i}{\sum_{i=1987}^{2007}(y_i - \mu)^2 w_i}$$

where y_i denotes the observed cases, accumulated over September to December for the year i, $\hat{y}_i$ is the predicted cases for the given model, accumulated over September to December for the year i and μ is the 20 year mean of the observed cases accumulated between September and December. For Barmer, the formula remains the same, except the time period of the summation changes and becomes 1985 to 2005. The weights used for the computation of the skill measure are denoted by w_i, and the natural choice of these weights (for a particular model) is the inverse of the prediction variance for the year i for the model in question. In Table 2, the columns show the skill measures for different forecast methods, using both constant weights (corresponding to the ordinary regression model) and the weights given by the inverse prediction variance for the VSEIRS malaria model with rainfall as a covariate.

Evaluating forecast skill by likelihood

Measures of forecast skill based on weighted mean squared error are sensitive to the choice of weights (Table 2). Equal weights, corresponding to ordinary regression, do not match well the observed relationship between increasing cases and increasing variability. However, it is difficult to defend any particular choice of non-equal weights. A less arbitrary approach is to compare forecasts by their prediction likelihood, which measures the probability density that each forecast assigns to the outcomes that in fact occurred. We proceed to give a formal description of this method. Each of the forecast rules $j = 1, \ldots, 4$ has a corresponding prediction density for a_i given the information available at the time of the forecast. In other words, rule j asserts that a_i is a realization of a random variable with a probability density function $f_{ij}(a)$. We suppress all dependences of f_{ij} on $a_1, \ldots, a_{i-1}$ or $r_1, \ldots, r_i$ or other information included in the forecast. Specifically,

$$\begin{aligned} f_{i1}(a) &= \frac{1}{\sqrt{2\pi V_i^{(1)}}} \exp\left\{-\left(\hat{a}_i^{(1)}\right)^2 / 2V_i^{(1)}\right\}, \\ f_{i2}(a) &= p_i \psi(a \mid \mu_1, s) + (1 - p_i)\psi(a \mid \mu_2, s) \end{aligned} \tag{S19}$$

where $\psi(\cdot \mid \mu, s)$ is the density of a Negbin(μ, s) random variable. The prediction density of a_i for the mechanistic models was calculated by applying kernel density estimation to the Monte Carlo forecast ensemble $\{\hat{a}_i^{[n]}, n = 1, \ldots, N\}$ as illustrated in Fig. S4. Kernel smoothing was carried out using the `kernel.smooth` function in R. The full prediction likelihood for method j is then

$$f^{(j)}(a_1, \ldots, a_n) = \prod_{i=1}^{n} f_{ij}(a_i).$$

These likelihoods should not be compared with the likelihoods for the complete monthly data. They are, however, proper likelihoods for the aggregated data $a_1, \ldots, a_n$ and so can properly be compared to each other by standard AIC techniques or likelihood ratio tests.

Supplementary Results

To investigate the likelihood function, we carried out iterated filtering starting at hundreds of randomly chosen initial conditions. Iterated filtering is only theoretically guaranteed to converge to a local maximum of the likelihood function, so diverse starting points are required both in theory and in practice to explore the parameter space. Table S1 shows the likelihood of the fitted models for Barmer and Kutch districts.

We also fitted a non-mechanistic SARIMA model for comparison. For both districts, the VSEIRS model with rainfall is favored, and the parameter estimates for this model are shown in Tables S3 and S4 for Kutch and Barmer respectively. We then investigated in more detail how the rainfall covariate facilitates the model's explanation of the data. Some results, extending those presented for Kutch in the main text, follow. Figures S5A and S5B show the simulations from the fitted values for the VSEIRS models with and without rainfall respectively, for Barmer district. It can be seen that in the absence of rainfall, the model fails to capture any of the large peaks present in the data. Also the general interannual variability of the peaks is better captured when rainfall is included. This is consistent with our results for Kutch. As noted for Kutch, these simulations are not next step predictions but twenty year trajectories starting from initial conditions in 1985. Figures 1 and S5 show that inclusion of rainfall in the model helps us capture the observed correlation in the data. Figure S6 shows both profiles of the duration of immunity for the VSEIRS model with and without rainfall. The estimated 95% confidence interval indicates a short duration of immunity for the model with rainfall in Barmer and Kutch. Figures S6A and S6B show the profile plot for the model with and without rainfall for Barmer and Kutch respectively. The duration of immunity is identified poorly for Barmer, and is much longer for Kutch, when the model without rainfall is considered. Figures S9A and S9B show the simulations from the fitted values for an 'intermediate' VSEIRS model for Kutch and Barmer districts respectively. This intermediate model consists of an VSEIRS model with rainfall, but with the parameters fixed in the values of the VSEIRS model without rainfall (i.e a model with long immunity), fitting only rainfall and noise parameters. It can be seen that when immunity is long, the inclusion of rainfall can not reproduce neither interanual variability nor outbreaks intensity.

References

1. Bretó C, He D, Ionides EL, King AA (2009) Time series analysis via mechanistic models. Annals of Applied Statistics 3: 319–348.

2. He D, Ionides EL, King AA (2010) Plug-and-play inference for disease dynamics: Measles in large and small towns as a case study. J R Soc Interface 7: 271–283.

3. Protter P, Talay D (1997) The Euler scheme for Lévy driven stochastic differential equations. The Annals of Probability 25: 393–423.

4. Jacod J (2004) The Euler scheme for Lévy driven stochastic differential equations: Limit theorems. Annals of Probability 32: 1830–1872.

5. Kloeden PE, Platen E (1999) Numerical Soluion of Stochastic Differential Equations. New York: Springer, 3rd edition.

6. Alonso D, Pascual M (2009). Enviromental-induced bifurcations in a malaria transmission model. In preparation.

7. Wearing HJ, Rohani P, Keeling MJ (2005) Appropriate models for the management of infectious diseases. PLoS Med 2: e174.

8. Lloyd AL (2001) Realistic distributions of infectious periods in epidemic models: changing patterns of persistence and dynamics. Theoretical Population Biology 60: 59–71.

9. Ionides EL, Bretó C, King AA (2006) Inference for nonlinear dynamical systems. Proc Natl Acad Sci USA 103: 18438–18443.

10. Ionides EL, Bhadra A, King AA (2009). Iterated filtering. Submitted for publication, and available online at. URL `http://arxiv.org/abs/0902.0347`.

11. Doucet A, de Freitas N, Gordon NJ, editors (2001) Sequential Monte Carlo Methods in Practice. New York: Springer.

12. Cappé O, Godsill S, Moulines E (2007) An overview of existing methods and recent advances in sequential Monte Carlo. Proceedings of the IEEE 95: 899-924.

13. King AA, Ionides EL, Pascual M, Bouma MJ (2008) Inapparent infections and cholera dynamics. Nature 454: 877–880.

14. King AA, Ionides EL, Bretó CM, Ellner S, Kendall B (2009) pomp: Statistical inference for partially observed Markov processes. URL `http://pomp.r-forge.r-rproject.org`.

15. Cazelles B, Chavez M, Constantin de Magny G, Guégan J, Hales S (2007) Time-dependent spectral analysis of epidemiological time-series with wavelets. J R Soc Interface 4: 625-636.
